# Slow bleaching of water-insoluble brown carbon from biomass burning: Implication for direct radiative effect

**DOI:** 10.1126/sciadv.aef9320

**Published:** 2026-08-21

**Authors:** Wenli Liu, Xinchun Xie, Lijuan Li, Rui Wang, Xin Zhang, Xinye Luo, Yuemei Han, Yuzhong Zhang, Mikinori Kuwata

**Affiliations:** ^1^ Department of Atmospheric and Oceanic Sciences, Laboratory for Climate and Ocean-Atmosphere Studies, Peking University, Beijing, China.; ^2^ Sichuan Academy of Eco-Environmental Sciences, Chengdu, Sichuan, China.; ^3^ College of Environmental and Resource Sciences, Zhejiang University, Hangzhou, Zhejiang, China.; ^4^ Key Laboratory of Coastal Environment and Resources of Zhejiang Province, School of Engineering, Westlake University, Hangzhou, Zhejiang, China.; ^5^ Institute of Advanced Technology, Westlake Institute for Advanced Study, Hangzhou, Zhejiang, China.; ^6^ Yixing Transportation Comprehensive Administrative Law Enforcement Brigade, Wuxi, Jiangsu, China.; ^7^ Key Laboratory of Aerosol Chemistry and Physics, State Key Laboratory of Loess Science, Institute of Earth Environment, Chinese Academy of Sciences, Xi’an, China.; ^8^ Chongqing Institute of Green and Intelligent Technology, Chinese Academy of Sciences, Chongqing, China.; ^9^ School of Human Settlements and Civil Engineering, Xi’an Jiaotong University, Xi’an, China.

## Abstract

Biomass burning organic aerosol (BBOA) is a major source of atmospheric brown carbon (BrC), which contributes to climate through solar radiation absorption. Chemical aging (bleaching) of BrC diminishes light absorption over time. Although recent modeling studies have emphasized the need to account for bleaching when assessing BrC’s climate impacts, a parameterization representing the entire BBOA matrix has been lacking. Here, we develop a bleaching parameterization for the whole BrC in BBOA based on laboratory experiments that systematically varied temperature and relative humidity. The bleaching timescale increases under low-humidity and low-temperature conditions, likely due to enhanced aerosol viscosity. Implementing this parameterization in a global model increases the simulated direct radiative effect (DRE) of BrC by 1.5 to 2 times relative to previous estimates, with associated uncertainties exceeding 10% of the total organic aerosol DRE. The present scheme particularly shows elevated fresh BrC concentrations in boreal regions, suggesting that bleaching dynamics may influence not only radiative forcing but also snow darkening effects.

## INTRODUCTION

Atmospheric aerosol particles strongly influence Earth’s energy balance by absorbing and scattering solar radiation (*1*). Light-absorbing aerosols such as dust and black carbon (BC) mostly contribute to atmospheric warming, whereas inorganic salts, including sulfate and sea spray, mainly scatter sunlight and exert a cooling effect (*2*). Organic aerosol (OA) constitutes a large fraction (typically 20 to 90%) of submicrometer particles in the atmosphere (*3*, *4*). Most OA components primarily scatter ultraviolet (UV) and visible light (*5*). In contrast, a subset of OA known as brown carbon (BrC) absorbs solar radiation (*6*). Both BC and BrC are therefore classified as carbonaceous aerosols (*6*).

Although BrC and BC both absorb light, their optical characteristics differ substantially. The absorption Ångström exponent (AAE) of BC is approximately unity, reflecting its graphite-like structure and wavelength-independent absorption (*6*). In contrast, BrC exhibits higher AAE values, typically between 1.5 and 10 (*7*), indicating stronger absorption in the UV and short-wave visible range (e.g., violet and blue). This wavelength dependence arises from molecular structures such as conjugated double bonds and nitroaromatics (*6*). Model simulations have estimated the direct radiative effect (DRE) of BrC to be approximately 0.10 W m^−2^ (*8*).

BrC originates from both primary emissions and secondary formation through atmospheric chemical reactions (*9*). Biomass and biofuel combustion represent dominant primary sources, contributing an estimated 50 to 70% of total global BrC (*8*, *10*). Biomass burning organic aerosol (BBOA) consists of water-soluble organic matter (WSOM) and water-insoluble organic matter (WISOM) components (*6*). Laboratory combustion studies have shown that WISOM dominates freshly emitted BBOA (*11*–*13*) and exhibits stronger light absorption per unit mass than WSOM (*14*, *15*). Consequently, WISOM is expected to be a key contributor to BrC light absorption in freshly emitted BBOA (*16*). A field study in Alaska indicated that WISOM is the dominant contributor to light absorption at 365 nm (*17*). A recent laboratory experiment demonstrated that WISOM exerts a substantially stronger contribution to visible light absorption than WSOM (*18*).

Field and laboratory studies have consistently observed photochemical degradation/bleaching of BrC, which decreases its light absorption efficiency over time (*19*–*22*). A synthesis investigation of wildfires in North America suggests that BrC typically exhibits an e-folding lifetime of approximately 1 day (*21*). The order of the timescale is comparable with measurements in other regions of the world such as Europe, the Amazon, South Asia, and East Asia (*19*, *23*–*25*). Most atmospheric models therefore assume a fixed bleaching timescale of around 1 day for BrC, independent of environmental conditions such as temperature and relative humidity (RH) (*10*, *26*, *27*). However, the chemical aging of OA is governed by particle-phase diffusion processes, which are sensitive to ambient conditions (*28*). Thus, the assumption of a constant bleaching timescale may not accurately represent atmospheric variability.

Recent studies have revealed that OA can transition into highly viscous or even glassy states under cold and dry environments (*29*–*31*). In such states, diffusion of reactive species within the particle phase becomes slower (*28*, *32*–*34*). When OA approaches the glass transition point, chemical aging processes may effectively cease on atmospheric timescales (*35*, *36*). Because viscosity increases as temperature and RH decrease, the bleaching of BrC in BBOA is expected to slow down considerably in the upper troposphere and polar regions. Schnitzler *et al.* (*31*) demonstrate that WSOM from BBOA can exhibit photobleaching lifetimes extending from months to years under such conditions. Consideration of the temperature and humidity dependences in BrC bleaching process reduces the gap between the field and modeling results (*37*). However, WSOM is not considered a dominant contributor to BrC in BBOA, as indicated by several recent studies (*12*, *17*, *18*).

The viscosities of hydrophilic (∼WSOM) and hydrophobic (∼WISOM) fractions of BBOA were independently investigated following the occurrence of liquid-liquid phase separation (*38*). The results demonstrate that the viscosity of the hydrophilic phase is governed by both temperature and RH, whereas that of the hydrophobic phase is primarily controlled by temperature. These findings highlight the need to better constrain BrC bleaching kinetics across the full chemical complexity of BBOA, which nevertheless remains poorly understood (*9*, *16*, *39*). Consequently, recent modeling studies conducted sensitivity analysis of BrC chemical aging as a function of altitude, while detailed parameterizations and mechanistic understanding of the underlying physicochemical processes remain limited (*27*).

To address this knowledge gap, we conducted a series of controlled ozonolysis experiments on BBOA as a function of temperature and RH. The BBOA was generated by smoldering wheat straw and collected on Teflon filters with carefully controlled particle mass loadings, enabling quantitative comparison across conditions. After ozone exposure, the samples were solvent extracted and analyzed using a liquid waveguide capillary cell (LWCC) and ultrahigh-performance liquid chromatography–diode array detector–high-resolution Orbitrap mass spectrometry (UHPLC-DAD-HRMS). Additional experiments on WSOM and WISOM fractions were performed to isolate the contributions of each component. Experimental results of the whole BBOA sample were subsequently parameterized for global climate models. Model simulations showed that accounting for the presence and slower bleaching of WISOM substantially enhances BrC’s DRE, particularly in the boreal region. These findings highlight the critical role of particle viscosity and environmental conditions in determining the radiative impacts of BrC.

## RESULTS

### Chemical characteristics of BBOA

**Fig. 1.**
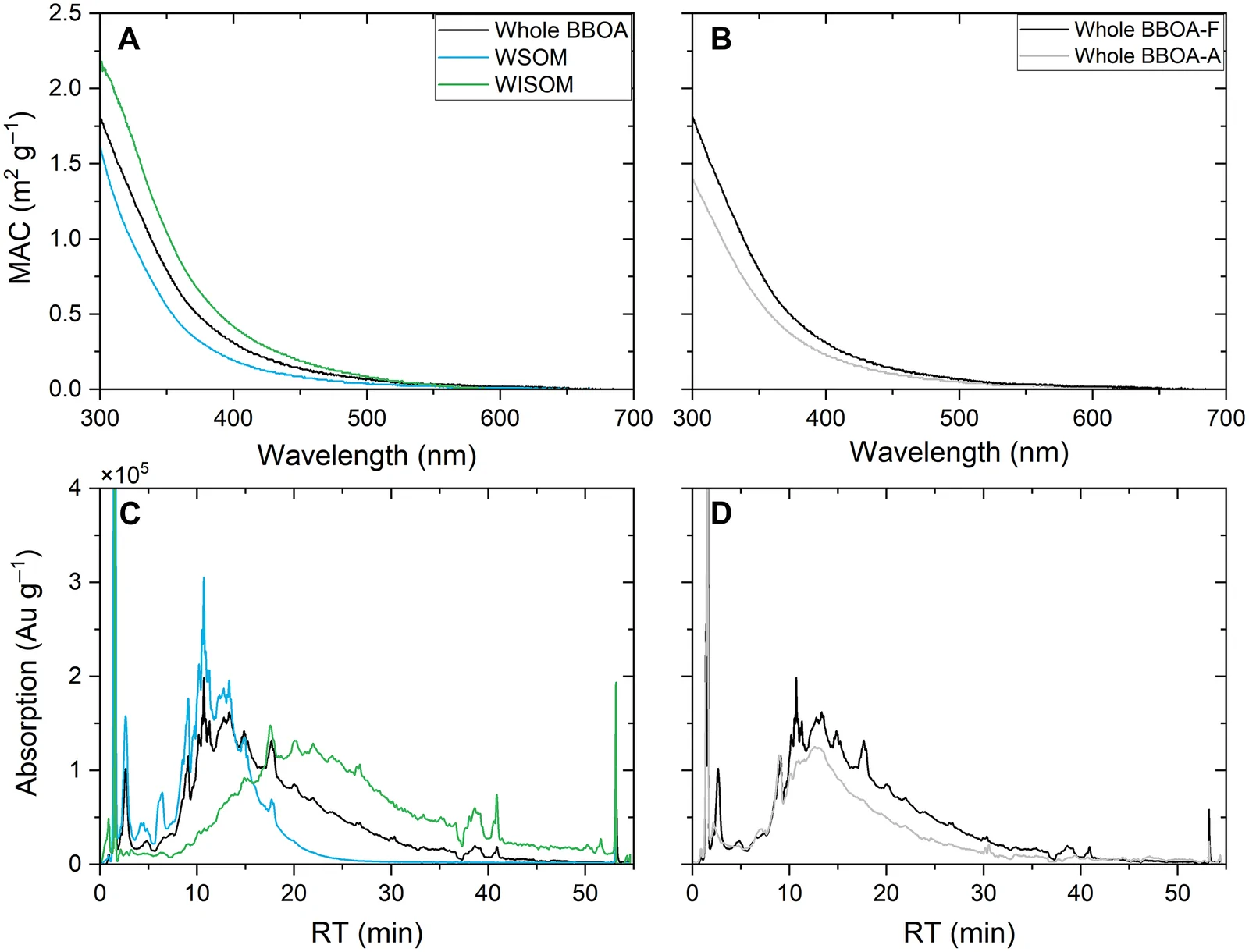
Light absorption properties of BBOA samples. MAC measured by the LWCC for (**A**) fresh samples (whole, WSOM, and WISOM fractions of BBOA), (**B**) fresh (-F) and aged (-A) samples of the whole BBOA. UHPLC-DAD chromatograms for (**C**) the fresh samples and (**D**) the fresh and aged samples of the whole BBOA. The experimental condition for aging was 9 ppm of ozone (8 hours of exposure), *T* = 298 K, RH = 85%. RT denotes retention time.

Figure 1 presents the comparisons of the optical properties of each fraction of fresh BBOA (Fig. 1, A and C), as well as the whole BBOA sample before and after chemical aging (Fig. 1, B and D). Both LWCC (Fig. 1, A and B, and fig. S1) and UHPLC-DAD (Fig. 1, C and D, and fig. S2) data are provided to illustrate these properties. Across the full wavelength range, the mass absorption coefficient (MAC) of WISOM was consistently higher than that of WSOM, in agreement with previous studies on fresh BBOA (*14*, *15*). The corresponding AAE values for WSOM, WISOM, and the whole BBOA sample were 7.4, 5.7, and 6.2, respectively. UHPLC-DAD analysis revealed that WSOM eluted predominantly at shorter retention times (<15 min), whereas WISOM species were mostly eluted at longer retention times (>15 min) (Fig. 1C and fig. S2). This confirms that water-soluble fractions are more polar than water-insoluble components.

The formation of new light-absorbing species was also observed (fig. S3). These newly formed BrC species were most prominent at retention times around 9 to 11 min before decreasing rapidly with further exposure. The enhancements peaked at approximately 1 hour of ozone exposure. Overall, these observations indicate that ozonolysis of BBOA predominantly promotes bleaching rather than darkening.

**Fig. 2.**
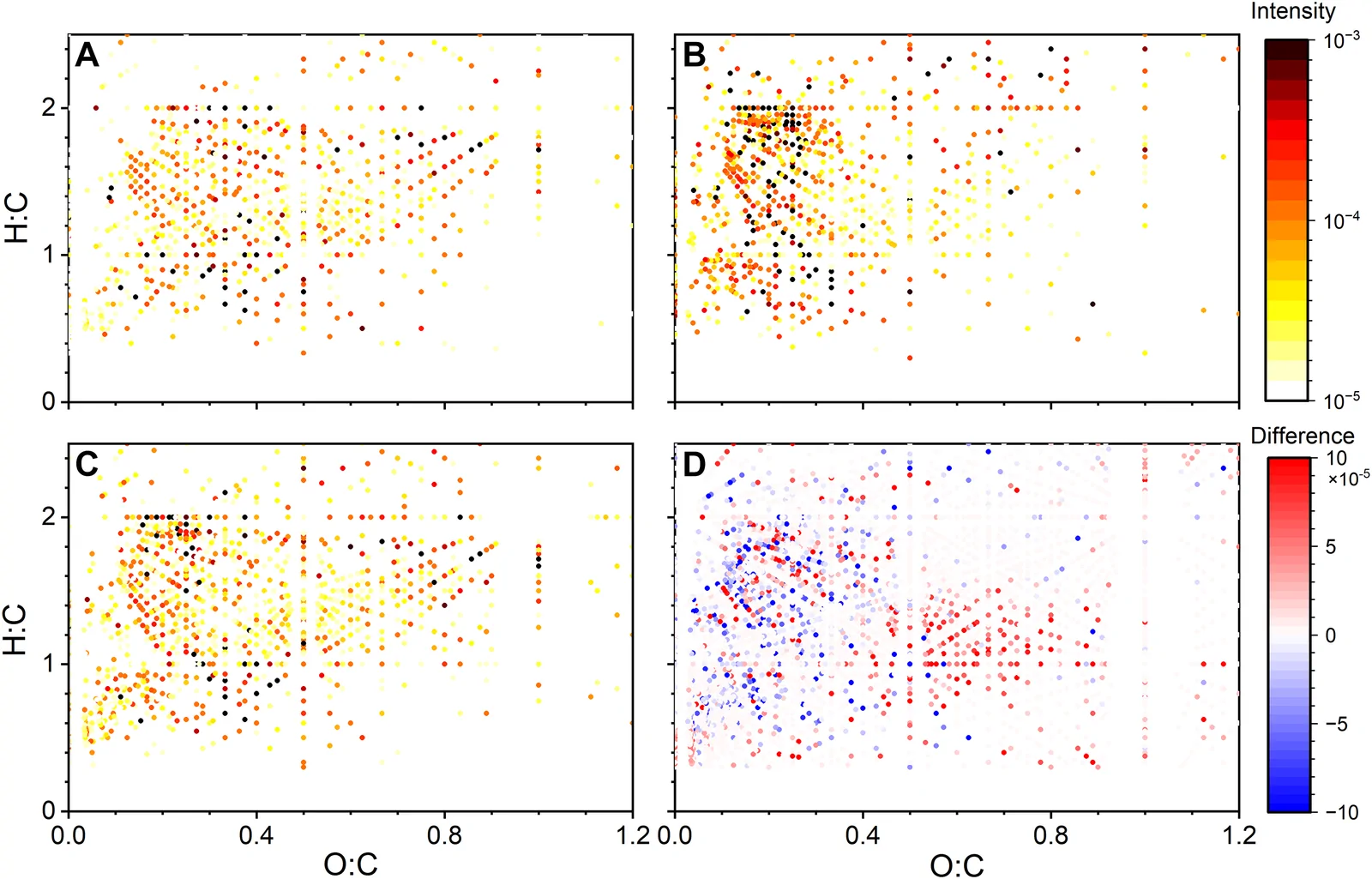
Van Krevelen diagrams for compounds in BBOA samples. Data for (**A**) fresh WSOM fraction, (**B**) fresh WISOM fraction, (**C**) fresh whole BBOA samples, and (**D**) the difference between the fresh and aged (8 hours of 9 ppm ozone exposure, *T* = 298 K, RH = 85%) whole BBOA samples. The normalized signal intensities for the ESI negative mode are shown.

Mass spectrometric analyses of fresh and oxidized BBOA samples are summarized in Fig. 2, fig. S4, and table S1. Van Krevelen diagrams (Fig. 2) show that most WSOM species were highly oxygenated (O:C > 0.3), although some exceptions were present. In contrast, WISOM species were generally less oxygenated (O:C < 0.3). Ozonolysis generally reduced the abundance of less-oxidized species, although some species with H:C = 1.2 to 2 exhibited enhanced abundance. Oxidation increased the abundance of highly oxidized species (O:C > 0.5), suggesting that part of WISOM may be converted into WSOM during chemical aging, considering the previous observations linking water solubility to elemental ratios (*40*).

Figure S5 presents the double bond equivalent (DBE) and molecular weight of identified chemical species. Less oxygenated and less hydrogenated species exhibited higher DBE values, whereas highly oxygenated, less hydrogenated species had smaller molecular weights. Comparison with Fig. 2 indicates that WSOM species generally had lower DBE and molecular weight than WISOM. Table S1 summarizes the average parameters for each sample. These trends align with previous findings that MAC is typically higher for less polar, larger, and less saturated compounds (*41*–*43*). Reductions in DBE and molecular weight after chemical aging therefore corroborate the observed bleaching behavior.

Glass transition temperatures (*T*_g_) were estimated using an empirical parameterization based on mass spectrometric data (table S1) (*44*). Estimated *T*_g_ values were 252, 285, and 255 K for WSOM, WISOM, and the whole BBOA sample, respectively. As noted by Koop *et al.* (*45*), molecular weight is the dominant factor influencing *T*_g_. The higher *T*_g_ of WISOM relative to WSOM reflects its larger molecular weight (table S1).

These results demonstrate that WISOM is an important contributor to BrC in BBOA, in agreement with previous studies (*9*, *16*–*18*, *39*). Oxidation of the whole BBOA sample suggests that bleaching predominantly occurred in the WISOM fraction. WSOM components exhibit higher O:C ratios than other fractions, implying greater hygroscopicity. The higher *T*_g_ and lower water content of WISOM and the whole BBOA sample suggest that their chemical aging may be slower than that of WSOM due to high viscosity. The kinetics of BrC bleaching from BBOA are discussed in the following section.

### Parameterizing bleaching lifetime of BrC

**Fig. 3.**
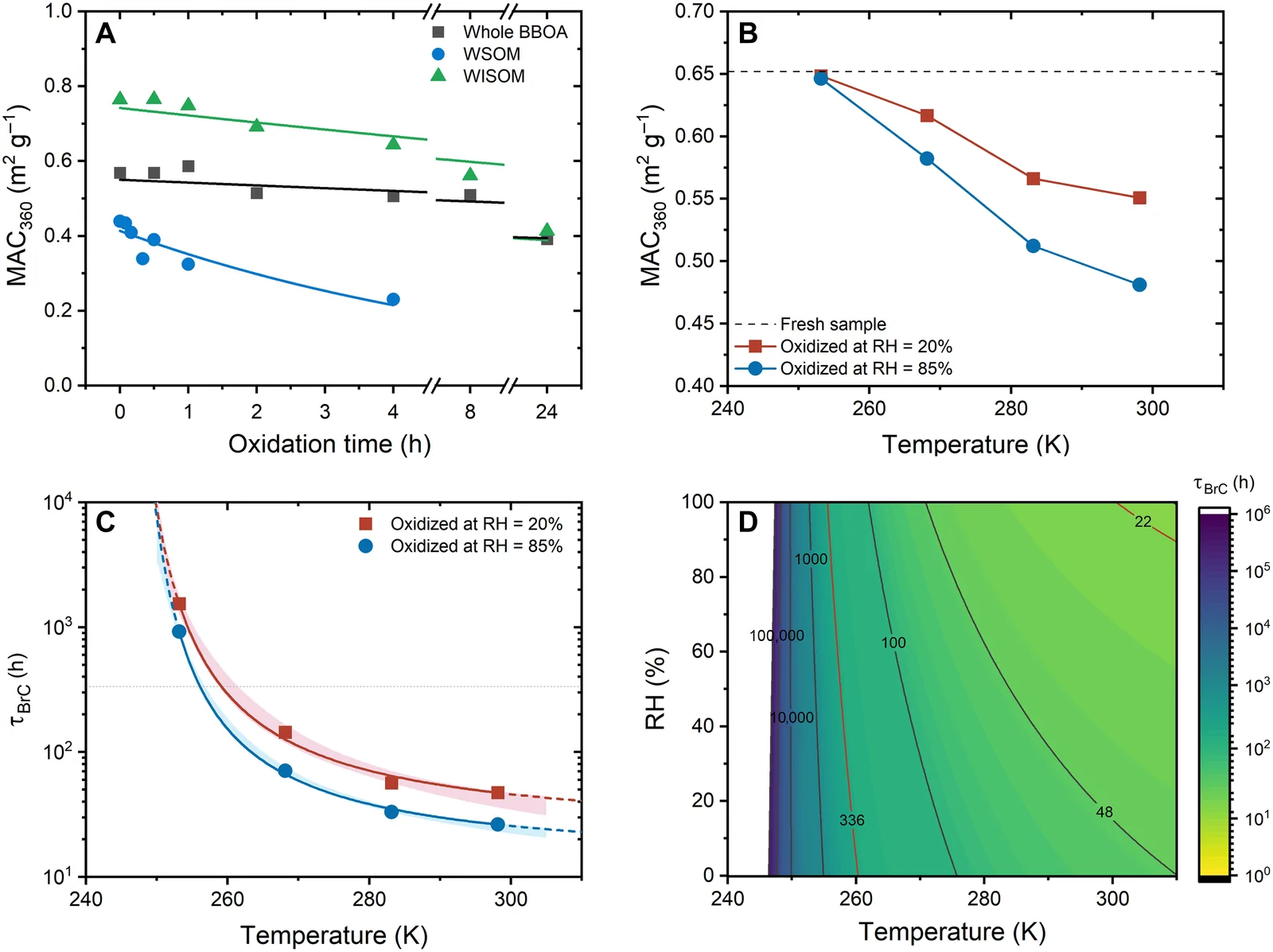
Bleaching of BrC in BBOA as a function of temperature and RH. (**A**) Reduction in MAC induced by ozone exposure at *T* = 293 K and RH = 85% for the whole, WSOM, and WISOM samples. Exponential fitting lines are also shown. (**B**) Changes in MAC for the whole BBOA sample following 8 hours of ozone exposure. The data are shown as a function of temperature and RH. (**C**) τ_BrC_ for the whole BBOA sample plotted as a function of temperature. Both the data for RH = 20 and 85% are shown. The experimental results (dots) and fitted curves for Eq. 4 are shown. Shaded areas illustrate the 95% confidence interval of the fitting calculated using the Monte Carlo method. (**D**) Developed parameterization for τ_BrC_ for the whole BBOA sample shown as a function of temperature and RH.

Figure 3A compares the reduction in MAC (λ = 360 nm) for WSOM, WISOM, and the whole BBOA samples following exposure to 9 parts per million (ppm) of ozone under conditions of *T* = 293 K and RH = 85%. The MAC of WSOM decreased to less than half of its initial value within 4 hours of ozone exposure, while less than 20% reduction was observed for WISOM and the whole BBOA samples during the same time period. For WISOM and the whole BBOA, approximately 24 hours of exposure were required for the MAC to decrease to half of its original value. Qualitatively similar behavior was observed across other wavelengths (fig. S6).

Figure 3B shows the MAC (λ = 360 nm) values after 8 hours of ozone exposure (9 ppm) for the whole BBOA sample as a function of temperature and RH. The bleaching process was accelerated under conditions of high temperature and RH, whereas ozonolysis of BBOA was strongly suppressed under low temperature and RH. The bleaching lifetimes (τ_BrC_) were obtained by phenomenologically fitting the experimental results for MAC (λ = 360 nm) using an exponential function

$$\text{MAC}\;\left(t\right)={\text{MAC}}_{0}e^{-\frac{t}{\tau_{\text{BrC}}}}$$ (1)

At *T* = 293 K (RH = 85%), the τ_BrC_ values were 6 and 37 hours for WSOM and WISOM, respectively, while the corresponding value for the whole BBOA sample (*T* = 298 K, RH = 85%) was 26 hours. These results confirm that WSOM exhibits higher chemical reactivity than WISOM and the whole BBOA sample. The range of τ_BrC_ obtained in this study is comparable to values reported from both aircraft and ground-based field observations, which typically span 0.5 to 5 days (table S2) (*19*, *21*, *24*, *25*, *46*).

The obtained τ_BrC_ values for the whole BBOA are summarized in Fig. 3C and table S3. The effect of humidity on τ_BrC_ was limited to a factor of a few (e.g., τ_BrC_ = 48 and 26 hours at RH = 20 and 85% for *T* = 298.15 K). However, the impact of temperature was much greater, varying τ_BrC_ by orders of magnitude (τ_BrC_ = 1.5 × 10^3^ hours at RH = 20% and *T* = 253.15 K). The observed increase in τ_BrC_ under low-temperature conditions is likely attributed to enhanced viscosity, consistent with previous studies on the chemical aging of OA (*31*, *33*, *34*).

A parameterization of τ_BrC_ for the whole BBOA sample was developed as a function of temperature and RH (text S1-1). The approach used a resistor model that describes diffusion-limited reactions (*31*, *47*)

$$\tau_{\text{BrC}}=\frac{\left(1-\frac{1}{\sqrt{e}}\right)2a\sqrt{{\left[\text{BrC}\right]}_{0}}}{3HP_{O_{3}}\sqrt{D_{O_{3}}k_{2}}}$$ (2)

Here, *a* represents particle diameter, [BrC]_0_ is the initial BrC concentration in the particle, *H* is Henry’s law constant, *P*_O3_ is the partial pressure of ozone, *D*_O3_ is the diffusion coefficient of ozone in BBOA, and *k*_2_ is the second-order rate constant for the reaction. As discussed by Schnitzler *et al.* (*31*), *H*(*k*_2_/BrC_0_)^1^/^2^ shows only weak temperature dependence, suggesting that the temperature and humidity dependence of τ_BrC_ primarily arises from variations in *D*_O3_.

The value of *D*_O3_ was parameterized as a function of temperature and humidity following the empirical formulation of Zobrist *et al.* (*48*) for the sucrose-water system, considering the change in particle viscosity with temperature

$$\text{ln}D=-\left(A+\frac{B}{T-T_{0}}\right)$$ (3)

where *A*, *B*, and *T*_0_ are empirically fitted parameters dependent on RH. By combining Eqs. 2 and 3, the following expression was obtained

$$\tau_{\text{BrC}}=C{\left(e^{A+\frac{B}{T-T_{0}}}\right)}^{\frac{1}{2}}$$ (4)

Here, *C* is a variable that depends on RH. The optimized parameters for Eq. 4 are summarized in table S4. The optimized parameters were robust against data uncertainty; details of the sensitivity analysis are provided in the Supplementary Materials (text S1-2 and fig. S7).

Schnitzler *et al.* (*31*) assumed *a* = 150 nm and *P*_O3_ = 35 parts per billion (ppb) to estimate τ_BrC_ under ambient conditions. In the present study, particles were exposed to ozone on Teflon filters, making direct estimation challenging. Subramanian *et al.* (*49*) demonstrated, using scanning electron microscopy, that BBOA tends to coat filter fiber rather than simply accumulate on it, implying that direct estimation of *a* is difficult. Our experiments obtained τ_BrC_ = 6.3 hours for WSOM from BBOA at *T* = 293 K (RH = 85%), while the corresponding value reported in Schnitzler *et al.* (*31*) was 4 hours. Assuming comparable chemical and physical properties of WSOM in both studies, this agreement suggests that the effective ratios of particle size and oxidant concentration (i.e., *a*/*P*_O3_) were similar. Therefore, the parameterization for τ_BrC_ was developed directly from experimentally obtained data. Particle mass loading on filter samples was kept within a similar range across WSOM, WISOM, and whole BBOA experiments (table S5). The resulting fit reproduced the experimental data well (Fig. 3C).

Figure 3D shows the parameterized τ_BrC_ for the whole BBOA sample as a function of atmospheric temperature and RH, along with reference lines at 22 and 336 hours. In global models, τ_BrC_ of around 1 day is commonly assumed (*10*, *26*). On the other hand, 336 hours (2 weeks) corresponds to a typical tropospheric lifetime of aerosol particles. The parameterization shows that τ_BrC_ varies by orders of magnitude with temperature but only by a factor of a few with humidity, consistent with the experimental data. The results also suggest that τ_BrC_ could exceed the atmospheric lifetime of the particles under cold conditions (*T* < ∼260 K).

Figure S8 compares τ_BrC_ values calculated using the parameterization by Schnitzler *et al.* (*31*) for WSOM from BBOA and the present study for whole BBOA particles. Their model exhibits stronger humidity dependence and weaker temperature sensitivity than the present parameterization. These differences likely reflect variations in hygroscopicity. The low-temperature hygroscopicity tandem differential mobility analyzer (Low-T HTDMA) measurements showed growth factors (GFs) of 1.15 and 1.10 for WSOM at 293 K (RH = 85%) and 253 K (RH = 80%), respectively, whereas those for WISOM were 1.04 and 1.01 (fig. S9). The greater water uptake of WSOM lowers viscosity at high RH, enhancing *D*_O3_. In contrast, such plasticizing effects are minimal for the whole BBOA sample due to its larger WISOM fraction. The comparison between the parameterizations developed from the whole BBOA sample (this study) and those based on WSOM (*31*) suggests that temperature may play a more dominant role than humidity in governing τ_BrC_.

Figure S10 compares the global distributions of τ_BrC_ calculated using the two parameterizations at 0, 2, and 8 km above the surface. In tropical fire regions (e.g., the Amazon, central Africa, and Southeast Asia), two parameterizations yield similar τ_BrC_ values. However, notable differences appear in boreal regions such as Alaska and Siberia. The discrepancy is attributed to lower temperatures (∼265 K) and moderate humidity (∼70%) in these regions.

### Modeling and estimation of BrC radiative impact

**Fig. 4.**
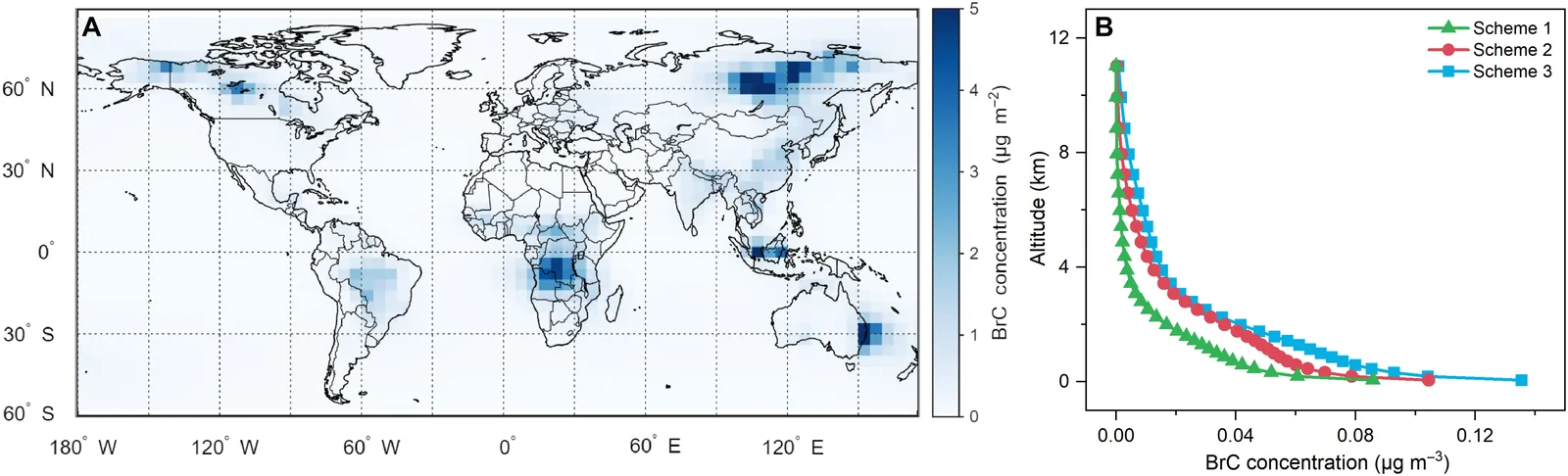
Model-simulated global and vertical distribution of BrC concentration. (**A**) Global distribution of fresh BrC column concentration using the parameterization developed by the present study. (**B**) Vertical profiles of fresh BrC concentration (global average) calculated by the model using τ_BrC_ of default setting (scheme 1), Schnitzler *et al.* (*31*) (scheme 2), and the present study (scheme 3).

Figure 4A summarizes the global distribution of column concentration for fresh BrC in whole BBOA estimated using GEOS-Chem, incorporating the parameterization developed in this study (Fig. 3D, scheme 3). For comparison, the corresponding results for the default setting (scheme 1, τ_BrC_ ∼ 1 day) and the parameterization by Schnitzler *et al.* (*31*) (scheme 2) are shown in fig. S11. Scheme 1 produced the lowest fresh BrC concentrations on the global scale among the three schemes, as τ_BrC_ is likely longer than 1 day across most regions of the world. Xie *et al.* (*37*) compared the global simulated water-soluble BrC with aircraft observations (ATom Mission). The results showed that the default simulation underestimated light absorption by more than an order of magnitude. Incorporating the parameterization of Schnitzler *et al.* (*31*) largely resolved this discrepancy, demonstrating that experiment-based parameterizations yield more realistic model outputs, at least for the water-soluble fraction.

The differences in fresh BrC column concentrations between the two parameterizations and the present one are summarized in fig. S12. Because the parameterization developed in this study is less sensitive to RH than that for WSOM (fig. S8), the simulated fresh BrC concentrations were lower in relatively dry regions, such as eastern Australia and southwestern Africa. In contrast, higher fresh BrC concentrations were predicted in the boreal regions (Siberia, Alaska, and Canada), reflecting the strong temperature dependence of τ_BrC_. The corresponding vertical distributions are shown in Fig. 4B and fig. S13, indicating that the parameterization developed here produced the highest fresh BrC concentrations throughout the troposphere. The higher abundance of BrC predicted by scheme 3 compared with scheme 2 in the upper troposphere is likely driven by the relatively large, RH-independent τ_BrC_ values estimated in scheme 3.

Table 1 and fig. S14 compare the DRE of BrC estimated using the three different bleaching parameterizations. Globally, the DRE of BrC was estimated to be 0.059, 0.088, and 0.125 W m^−2^ for the three schemes, respectively. The differences in DRE were particularly pronounced in the boreal region, consistent with the spatial variations in BrC concentrations (fig. S11). Compared with the total DRE of OA (−0.42 W m^−2^) at the top of the atmosphere under all-sky conditions (*50*), these results suggest that the treatment of BrC bleaching can influence the DRE of OA by more than 10%. This finding underscores the importance of incorporating the bleaching processes of the WISOM fraction when evaluating the radiative impact of OA. DRE was not substantially affected by the injection height, except in the extreme case where 100% of emissions were confined within the planetary boundary layer (PBL; 0% above the PBL) (fig. S15 and table S6).

**Table 1.** Summary of model settings and calculated BrC DRE.

	τ_BrC_	DRE (W m^−2^)
**Scheme 1**	One day	0.059
**Scheme 2**	Follow Schnitzler *et al.* (*31*)	0.088
**Scheme 3**	Follow this study (whole BBOA particles)	0.125

There are several caveats regarding the developed parameterization and its application in global models. The experiments conducted in this study focused exclusively on ozonolysis. However, other atmospheric oxidants, including OH radicals, are also known to oxidize BBOA and contribute to BrC bleaching (*51*, *52*). In general, temperature exerts a similar influence on the chemical reactivity of organic compounds toward both ozone and OH radicals. Li *et al.* (*33*) reported that the reactive uptake coefficients of ozone and OH radicals on canola oil films remain consistently low at temperatures below the melting temperature (*T*_m_), whereas substantially higher values were observed at temperatures above *T*_m_. These findings indicate that the transition in chemical reactivity occurs at similar temperatures for both oxidants. Accordingly, we expect that BrC bleaching driven by various oxidants would exhibit comparable temperature and RH dependences, although differences in oxidant reactivity and atmospheric abundance may result in different τ_BrC_ values.

The study focused on BBOA generated from wheat straw smoldering. In general, oxidations of OAs are enhanced at elevated temperature and RH, although the exact oxidation kinetics depends on aerosol compositions. The T/RH dependence of the oxidation kinetics of OA has been interpreted in terms of reduced viscosity under higher T/RH conditions (*34*, *53*, *54*). Accordingly, the key concept of the present finding (i.e., temperature/RH-dependent BrC bleaching) is expected to be broadly applicable to other types of BBOA, although the specific values of τ_BrC_ may still be influenced to some extent by emission source characteristics.

The discussion presented in this study focuses solely on primary BrC oxidation from BBOA. In contrast, for secondary OA (SOA), BrC formation is known to occur, especially when reactions involving heteroatoms take place (*27*, *55*, *56*). Secondary aerosols such as sulfate and SOA may also internally mix with BBOA, altering the mixing state during atmospheric transport. These changes can influence both the phase state and chemical reactivity of the aerosol. To accurately simulate the climate impacts of BrC, such interactions need to be considered so that laboratory findings can be effectively incorporated into global models.

No assumptions were made regarding phase mixing or phase separation, as the parameterization was developed phenomenologically from experimental data. Liquid-liquid phase separation may influence the oxidation kinetics of BBOA, as exposure to gas-phase oxidants would primarily occur in the outer organic phase. Although this remains an important topic, only a limited number of pioneering studies have addressed it to date (*57*). Particle acidity and surfactant effects may further modify oxidation kinetics by influencing phase state and morphology. Acidity can promote or suppress liquid-liquid phase separation in the presence of organic acids, while surfactants may reduce phase separation between aqueous and organic phases (*58*, *59*). These factors are therefore expected to affect chemical reactivity through changes in particle morphology and are not explicitly represented in the present parameterization.

The agreement of τ_BrC_ for the WSOM sample with previous studies conducted on renebulized WSOM from pine wood combustion (*31*), as well as the comparable τ_BrC_ for the whole BBOA sample under room conditions with field observations (*19*, *23*, *46*), provides qualitative support for the experimental approach. However, we emphasize that these comparisons are not intended to represent a strict quantitative validation, but rather to demonstrate consistency in the order of magnitude across laboratory and field studies. Further studies focusing on the WISOM fraction and its role in phase state–dependent reactivity will be important for improving the mechanistic representation of BrC bleaching in atmospheric models.

## DISCUSSION

The present study demonstrated that consideration of the whole components (i.e., WSOM and WISOM) extends the τ_BrC_ of BrC in BBOA, as shown through laboratory experiments. The prolonged τ_BrC_ of whole BBOA particles is likely governed by temperature-dependent viscosity change of the WISOM fraction, while the viscosity of the WSOM fraction is considerably influenced by water uptake. This extension of τ_BrC_ enhanced the estimated fresh BrC concentration, leading to an increased DRE of BrC, particularly in the boreal region.

An accurate estimation of BrC concentration is also essential for evaluating the snow darkening effect (SDE) (*60*, *61*). Brown *et al.* (*62*) reported that the land mean SDE of BrC could range from 0.021 W m^−2^ when bleaching is considered to 0.056 W m^−2^ when bleaching is neglected. For comparison, the corresponding value for BC was 0.057 W m^−2^. Deposition of BrC contributing to the SDE mainly occurs in high-altitude and high-latitude regions, where the parameterization developed in this study predicts longer τ_BrC_. These results highlight the importance of representing BrC bleaching processes for improving our understanding of climate forcing by biomass burning emissions.

Biomass burning is also a substantial source of polycyclic aromatic hydrocarbons (PAHs). The shielding effect provided by highly viscous OA has been shown to influence the atmospheric transport of PAHs to remote regions such as the Arctic (*29*, *63*). Combined with recent findings on the viscous nature of BBOA (*38*, *64*), the present study suggests that BBOA itself acts as a shield, extending the atmospheric chemical lifetimes of PAHs.

The influence of BrC on atmospheric photochemistry has also been discussed in recent literature. Modeling studies have shown that light absorption by BrC can reduce the photolysis rate of NO_2_, thereby affecting atmospheric oxidant concentrations (*10*, *37*). Furthermore, certain BrC compounds derived from BBOA can act as photosensitizers, potentially contributing to aerosol chemistry (*65*). Accurate estimation of global BrC distributions is therefore critical for understanding these processes. We suggest that both water-soluble and water-insoluble fractions of BBOA should be considered in future studies to achieve this goal.

## MATERIALS AND METHODS

### Preparation of BBOA samples

The experimental setup for biomass burning aerosol (BBOA) generation is illustrated in fig. S16. The configuration was similar to that used in our previous study (*66*). Approximately 2 g of sample (wheat straw powder) was placed in a ceramic crucible wrapped with a heating rope, with a thermocouple attached to the crucible surface. The temperature was maintained at 350°C using a PID controller (Model E5CC, Omron) for inducing smoldering. It typically required about 10 min to reach the target temperature.

The crucible was housed in a sealed stainless-steel chamber (50 liters) equipped with inlet and outlet ports for airflow. Zero air was continuously supplied to the chamber at a rate of 1.5 to 3 liters min^−1^, depending on the sampling conditions (table S7). The exhaust from the chamber was passed through a diffusion dryer containing activated charcoal to remove gas-phase species before particle collection.

BBOA particles were collected on polytetrafluoroethylene (PTFE) filters (47 mm, Pall Corporation) and borosilicate glass fiber filters (47 mm, Whatman Grade EPM 2000). PTFE filter samples were used for oxidation experiments on the whole BBOA samples. The BBOA-loaded PTFE filters were directly used for ozonolysis experiments to avoid potential artifacts associated with solvent extraction. Glass fiber filter samples were used to extract WSOM and WISOM fractions. The PTFE filters were weighed before sampling for gravimetric analysis. Glass fiber filters were prebaked at 500°C for 24 hours before use.

Sampling flow rates were controlled using mass flow controllers (MFCs). Sample collection began 15 min after the heater was turned on. The sampling durations and flow rates for all combustion experiments are summarized in table S7. After sampling, filters were stored in prebaked petri dishes covered with aluminum foil at −20°C until usage.

Particles collected on glass fiber filters were sequentially extracted with water and methanol. WSOM was obtained by sonicating the filters with 20, 15, and 15 ml of HPLC-grade water for 30 min. Subsequently, the filters were extracted with 20, 15, and 15 ml of methanol to obtain the WISOM fraction. The resulting extracts were filtered through 0.2-μm PTFE syringe filters (514-0070, VWR). The filters after extraction, as well as the final extracts, were nearly colorless, suggesting that most light-absorbing species had been effectively extracted (fig. S17). WSOM and WISOM particles were regenerated by nebulizing the corresponding solutions, and the resulting particles were collected on PTFE filters.

### Ozonolysis experiment

The setup for oxidation experiments is shown in fig. S18. BBOA particles collected on PTFE filters were exposed to ozone at a concentration of 9 ppm. Temperature and RH were controlled during the exposure experiments. Experimental parameters are summarized in table S5. The variables investigated included ozone exposure time (5 min to 24 hours), temperature (253 to 298 K), and RH (20, 55, and 85%). Ozone concentrations were monitored using an ozone analyzer (Model 49i, Thermo Fisher Scientific Inc.), and RH was measured with an RH sensor (Rongce, FG6010) and a dew point sensor (Model S-1M, Shinyei Technology Co. Ltd.).

### Sample analysis

Mass loadings of particles on the filters were quantified gravimetrically using an electronic microbalance (Model ME5-F, Sartorius, Göttingen, Germany). Each filter was extracted three times with a mixture of acetonitrile and water (volume ratio 9:1; total 9 g). The combined extract was sonicated for 10 min and filtered through a 0.2-μm nylon filter. The resulting sample solutions were concentrated under a nitrogen stream and reconstituted to 0.5 or 1 ml for chemical composition analysis.

Sample solutions were analyzed using an ultrahigh-performance liquid chromatography–diode array detector–high-resolution Q Exactive Orbitrap mass spectrometer (UHPLC-DAD-HRMS, Thermo Fisher Scientific, Germany). Eluates from the UHPLC were directed into the DAD for UV-Vis absorption measurements for the wavelength range of 190 to 800 nm. The samples were then ionized using an electrospray ionization (ESI) source and analyzed by HRMS in full-scan mode (mass/charge ratio = 50 to 750). Raw data were processed using Xcalibur (version 4.2.47) and MZmine (version 2.53). Additional details of the chemical analysis are provided in text S2 and Lin *et al.* (*67*).

An LWCC (Model 3100 Series, WPI) was used to direct measurement of the light absorption properties of whole samples. The LWCC covered a wavelength range of 230 to 730 nm, with an optical path length of 1 m. One milliliter of each diluted sample solution was injected for analysis. The measured MAC values agreed well with those integrated from UHPLC-DAD measurements in the 300- to 400-nm range (fig. S19).

### Hygroscopicity

Hygroscopicity measurements were conducted using a Low-T HTDMA as described in our previous study (*68*). Briefly, the system consisted of two differential mobility analyzers (DMAs) and two Shirasu porous glass (SPG) humidification tubes installed within a temperature-controlled area cooled by a freezer and a coolant circulation system. Before entering the low-temperature area, particles were dried (RH < 5%) and charge neutralized using an x-ray neutralizer (Model 3088, TSI). Monodisperse particles with a diameter of 100 nm selected by the first DMA were subsequently introduced into the SPG tubes for humidification. The second DMA was used to determine the particle size under humidified conditions. The hygroscopic GF was defined as the ratio of the humidified particle diameter to its corresponding dry diameter. Instrument calibration was performed using ammonium sulfate particles.

### Global model

The GEOS-Chem model coupled with the Rapid Radiative Transfer Model for Global Circulation Models (RRTMG) was used to simulate the concentration of BrC and its radiative impacts for the year 2019. Model details are provided in Xie *et al.* (*37*). Briefly, simulations were performed at a horizontal resolution of 4° by 5° with 47 vertical levels, driven by MERRA-2 meteorological fields. Biofuel and biomass burning emissions were based on Bond *et al.* (*69*) and GFED4s (*70*), with fresh aerosols treated as BrC. Approximately 35% of fire emissions were lofted above the PBL. Sensitivity tests were done with different emission fractions above PBL.

Three parameterization schemes for BrC bleaching lifetime were implemented in the model (Table 1). The simulation incorporating the τ_BrC_ parameterization developed in this study (scheme 3) was compared with the default setting (scheme 1, constant = ∼1 day) and the parameterization of Schnitzler *et al.* (*31*) (scheme 2). MAC values for biomass burning and biofuel aerosols at 365 nm were calculated as 1.3 and 1.2 m^2^ g^−1^, respectively, using Mie theory. Bleaching was assumed to reduce the MAC of aged BrC to one-quarter of that of fresh BrC.

## Supplementary Materials

### This PDF file includes:

Supplementary Text S1 and S2 Figs. S1 to S19 Tables S1 to S7 References
