# Supplementary material for: Slow bleaching of water-insoluble brown carbon from biomass burning: Implication for direct radiative effect
Source: Sci Adv. 2026 Aug 21;12(34):eaef9320. doi: 10.1126/sciadv.aef9320 (PMC13496173; doi:10.1126/sciadv.aef9320)
Supplement: Supplementary file 1 [file sciadv.aef9320_sm.pdf]

Science Advances
AAAS

Supplementary Materials for

Slow bleaching of water-insoluble brown carbon from biomass burning: Implication for direct radiative effect

Wenli Liu *et al.*

Corresponding author: Mikinori Kuwata, kuwata@pku.edu.cn

Sci. Adv. **12**, eaef9320 (2026)
DOI: 10.1126/sciadv.aef9320

This PDF file includes:

Supplementary Text S1 and S2
Figs. S1 to S19
Tables S1 to S7
References

Supporting Information Text

Text S1 Parameterization of τ_{BrC}

Text S1-1 Parameterization with four fitted parameters

The reaction rate between ozone and BBOA can be simplified using a resistor model, which considers multiple processes including diffusion, surface accommodation, and chemical reaction. Equation S1-1 describes the case in which the uptake of ozone in BBOA particles occurs in a thin shell below the particle surface (*31, 47*).

$$\sqrt{\frac{[BrC]_t}{[BrC]_0}} = 1 - \frac{3HP_{O_3}\sqrt{D_{O_3}k_2}}{2a\sqrt{[BrC]_0}}t \qquad \text{(S1-1)}$$

Where $[BrC]_0$ and $[BrC]_t$ are the BrC concentrations in particles at oxidation times of 0 and t, respectively. H is the Henry's law constant, and P_{O3} is the partial pressure of ozone. D_{O3} is the diffusion coefficient of ozone in the BBOA particle, k_2 is the second-order rate constant for the reaction, and a corresponds to the mode radius of the particles. t is the reaction time.

The bleaching lifetime can then be expressed by equation S1-2 following the e-fold definition.

$$\tau_{BrC} = \frac{\left(1 - \frac{1}{\sqrt{e}}\right)2a\sqrt{[BrC]_0}}{3HP_{O_3}\sqrt{D_{O_3}k_2}} \qquad \text{(S1-2)}$$

The temperature dependence of ozone's diffusion coefficient is governed by the Vogel-Fulcher-Tamman (VFT) equation, as expressed in equation S1-3 (*48*), where the fitting parameters A, B, and T_0 depend on both environmental temperature and RH:

$$lnD = -\left(A + \frac{B}{T - T_0}\right) \qquad \text{(S1-3)}$$

Equation S1-4 is obtained by substituting equation S1-3 into equation S1-2:

$$\tau_{BrC} = C\left(e^{A + \frac{B}{T - T_0}}\right)^{\frac{1}{2}} \qquad \text{(S1-4)}$$

where the parameter $C = \frac{\left(1 - \frac{1}{\sqrt{e}}\right)2a\sqrt{[BrC]_0}}{3HP_{O_3}\sqrt{k_2}}$. The fitted parameters are listed in Table S4.

Poke-flow experiments have demonstrated that particle viscosity exhibits an approximately linear negative correlation with RH/particle water content (*31*), indicating a positive relationship between the diffusion coefficient D and RH. The k_2 value in parameter C is also influenced by RH, as chemical reactivities of particles generally increase under high-humidity conditions (*32*). At a constant temperature, when RH increased from 20% to 85%, the chemical atmospheric lifetime of BBOA changed by approximately a factor of two. For simplification, the parameters A, B, C, and T_0 were assumed to be linear functions of RH. Fitting the experimental data to equation S1-4 yielded the following functions S1-5 to S1-8.

$$A = -0.00792 * RH(\%) + 21.29 \qquad \text{(S1-5)}$$

$$B = -0.1858 * RH(\%) + 99.129 \quad (S1\text{-}6)$$

$$T_0(K) = 0.0208 * RH(\%) + 241.738 \quad (S1\text{-}7)$$

$$C = -0.00000171 * RH(\%) + 0.000554 \quad (S1\text{-}8)$$

Text S1-2 Fitting with three parameters

T_0 in equation S1-3 is the Kauzmann temperature, for which the configurational entropy of the system vanishes (*71*). The T_0 value is slightly lower than the glass transition temperature T_g. The T_0 value is estimated with the parameterization developed by DeRieux *et al.* (*44*).

$$Tg = (n_C^0 + ln(n_C))b_C + ln(n_H)b_H + ln(n_C)ln(n_H)b_{CH} + ln(n_O)b_O + ln(n_C)ln(n_O)b_{CO}$$

where, C_0, b_C, b_H and b_O are fitted parameters from 300 compounds with multi-linear least squares analysis. n_C, n_H and n_O are the numbers of carbon hydrogen, and oxygen atoms. In this study, the HPLC-HRMS was employed to measure the chemical composition of BBOA in both ESI- and ESI+ modes. T_g values were estimated by the intensity averaged formula (Table S1). The average T_g value of the two modes, 238.95K, was approximated as T_0. The three-parameter equation (S1-9) was obtained and used to fit the experimental data.

$$\tau_{BrC} = C\left(e^{A+\frac{B}{T-238.95}}\right)^{\frac{1}{2}} \quad (S1\text{-}9)$$

The fitted parameters and results are shown in Table S4 and fig. S7. The discrepancy between the two parameterizations was within $\pm$20% for the temperature range of interest (fig. S7).

Text S1-3 Uncertainty estimation by the Monte-Carlo method

The Monte Carlo method estimated confidence intervals by generating synthetic datasets that mimicked outcomes of repeated experiments. First, the standard deviation of the relative residuals (σ) was calculated from the initial fit and measurement. Random noise ε was produced assuming a normal distribution with mean zero and standard deviation σ. For each simulation, ε was added to the measurement data, producing a new dataset that represents one possible realization of the experiment. Each synthetic dataset was refitted to obtain a set of parameters. After 5000 of simulations, the 2.5th and 97.5th percentiles of the collected parameter values formed the 95% confidence interval.

Text S2 BBOA measurements

Text S2-1 UHPLC-DAD-HRMS measurement

A 5 µL sample was eluted on a Thermo Hypersil Gold C18 column (100 × 2.1 mm, 1.9 µm particle size) at 30 °C. The mobile phase consisted of 0.05% formic acid in (A) pure water and (B) acetonitrile. The elution was performed at a flow rate of 0.2 mL min^{-1} for a total of 55 minutes: mobile phase B at 5% from 0 - 3 min, linearly increased to 30% from 3 - 10 min, then increased to 95% B from 10 - 45 min and maintained for 5 min, and finally decreased to 5% B within 1 min and maintained for 4 min.

The UHPLC-separated eluent was introduced into the DAD. A deuterium lamp and a tungsten lamp served as the ultraviolet and visible light sources, respectively, enabling absorbance measurements across a wavelength range of 190 - 800 nm.

The sample solutions were further introduced to a heated electrospray ionization source (ESI). Spray voltages for positive (ESI$^+$) and negative (ESI$^-$) modes were set to +4 kV and −3 kV, respectively. The Orbitrap operated in full-scan mode, with the quadrupole (QE) disabled. The measurable *m*/*z* range of the Orbitrap was 50 - 700, with a resolution of approximately 140,000 at *m*/*z* = 200.

Text S2-2 HRMS data analysis

Raw mass spectra from UHPLC-HRMS were processed using MZmine 2.53 for chemical composition identification. The noise level was set to 10,000, and Gaussian peak fitting was applied. The resolution was set to 140,000, with mass tolerances of 3 ppm and 2 ppm for positive and negative ion modes, respectively. For molecular identification, the number of atoms for C, H, O, N, and S was constrained to 1 - 40, 1 - 80, 0 - 50, 0 - 4, and 0 - 2, respectively *(67)*.

The identified compounds required further validation based on two criteria. First, peaks with signal intensities below ten times of the blank sample level were removed from the list. Second, the elemental ratios of the identified species were constrained to the following ranges: H/C (0.3 - 3), O/C (0 - 3), N/C (0 - 1.3), and S/C (0 - 0.8). Compounds outside these ranges were considered non-existent.

For data with completed species identification, elemental characteristics and the degree of unsaturation were calculated. Double Bond Equivalent (DBE) was calculated using equations S2-1 *(43)*.

$$DBE = 1 + \frac{2C - H + N}{2} \qquad (S2-1)$$

Text S2-3 DAD and LWCC data analysis

According to the Beer-Lambert law, the attenuation of light intensity through a sample is proportional to the concentration of light-absorbing substances and the optical path length. The mass absorption coefficient (MAC) at each wavelength was calculated using equation S2-3 for DAD *(52)*:

$$MAC(m^2 g^{-1}) = \frac{A(Au\ s) \times F(m^3\ min^{-1}) \times \ln(10)}{l(m) \times m(g) \times 60(s\ min^{-1})} \qquad (S2-3)$$

where *A* is the integrated peak area over elution time at a given wavelength. *F* is the HPLC flow rate (0.2 mL min^{-1}), and *l* is the optical path length of the DAD light pipe (7 mm). *m* is the mass of the injected sample, determined by precision weighing and the volumetric fraction of the injected solution.

For LWCC, MAC values were calculated using equation S2-4, where A is the measured absorbance, V is the injection volume (1 mL), and l is the optical path length of the LWCC (1 m):

$$MAC(m^2g^{-1}) = \frac{A(Au) \times V(m^3) \times \ln(10)}{l(m) \times m(g)} \qquad (S2-4)$$

The Ångström absorption exponent (AAE), which describes the wavelength dependence of the sample's light absorption capacity, was derived as the negative slope of the logarithmic plot of MAC versus wavelength in the 300 - 400 nm range, as expressed in equation S2-5:

$$AAE = -\frac{d(lnMAC_{\lambda})}{d(\ln\lambda)} \qquad (S2-5)$$

Figures

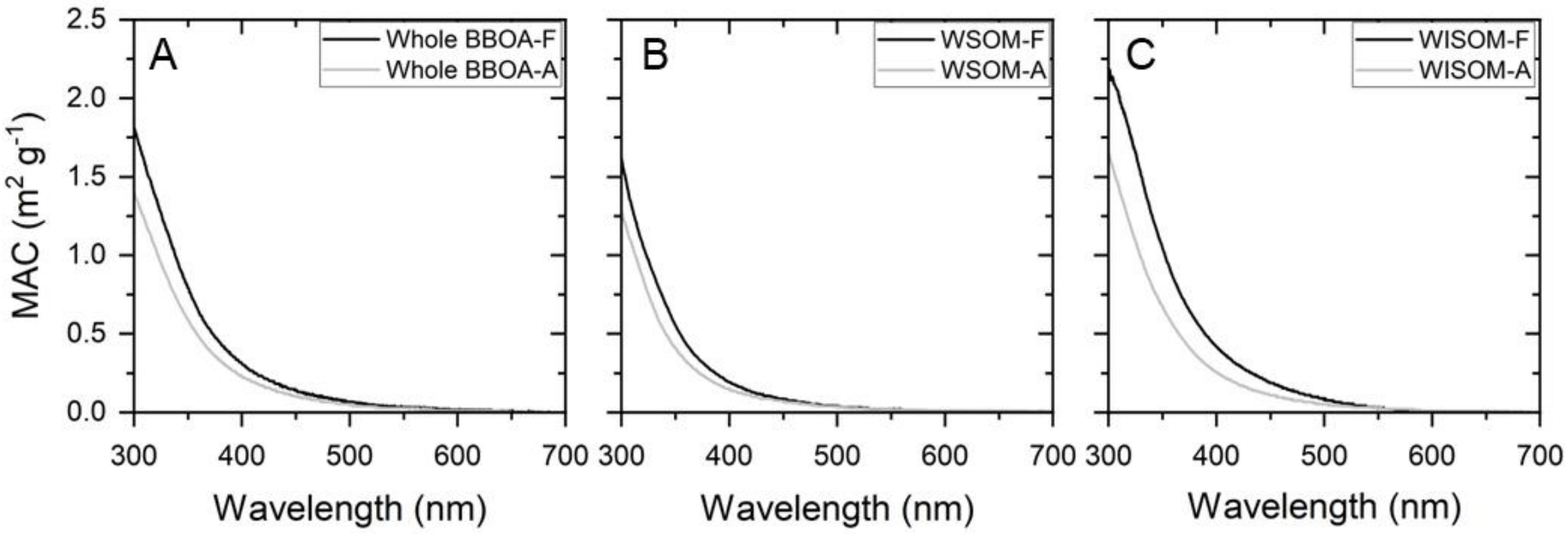

Fig. S1. MAC of BBOA samples measured by the LWCC. Data for (A) whole, (B) WSOM fractions, and (C) WISOM fraction of BBOA. Data for both the fresh (-F) and aged (-A) samples are shown. The oxidation conditions for the chemical aging were ozone exposure at 9 ppm for 8 (whole), 4 (WSOM) and 24 (WISOM) hours, T = 298 K (Whole) and 293 K (WSOM and WISOM), and RH = 85%. (A) is the same as Fig. 1B.

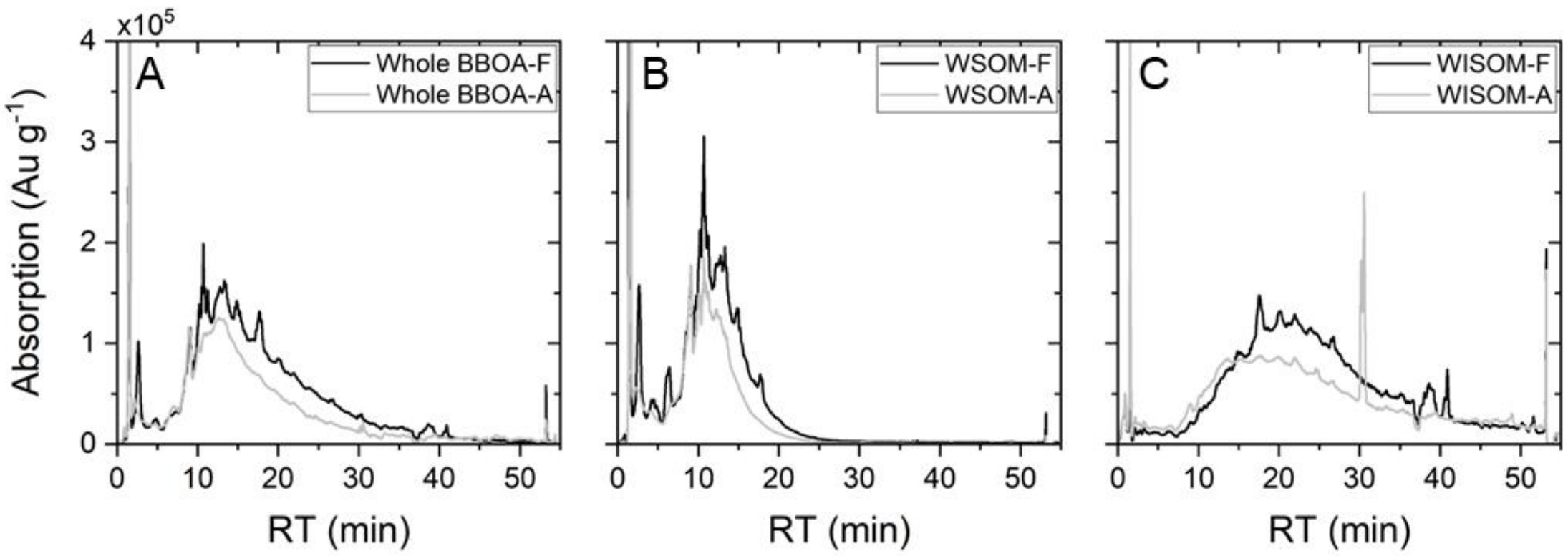

Fig. S2. UHPLC-DAD chromatograms for BBOA samples. Data for (A) whole, (B) WSOM fraction, and (C) WISOM fraction of BBOA samples. Data for both the fresh (-F) and aged (-A) samples are shown. The oxidation conditions for the chemical aging were ozone exposure at 9 ppm for 8 (whole), 4 (WSOM) and 24 (WISOM) hours, T = 298 K (Whole) and 293 K (WSOM and WISOM), and RH = 85%. (A) is the same as Fig. 1D.

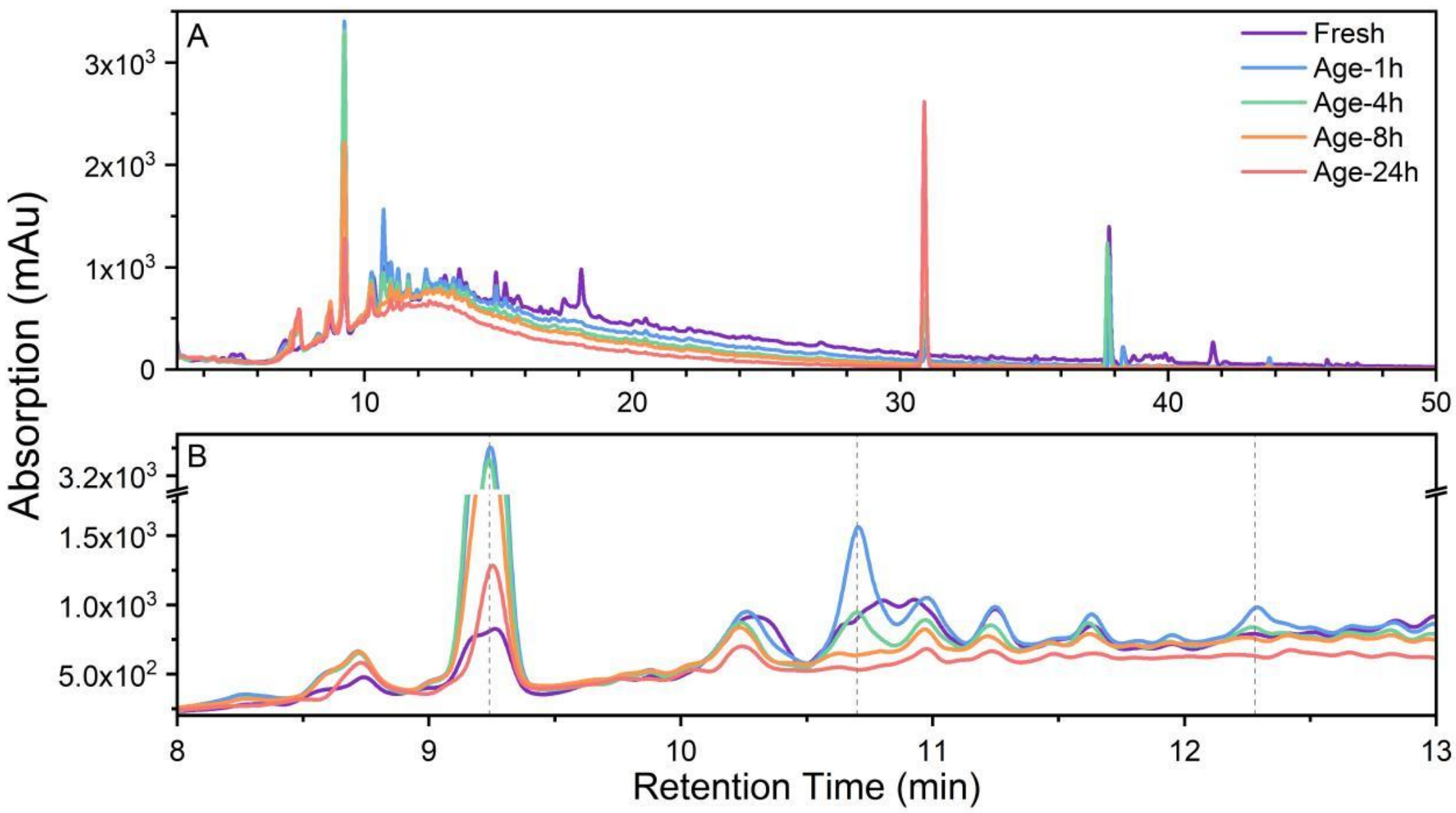

Fig. S3. Formation and loss of some chromophores suggested by the UHPLC-DAD. The data for the fresh, 1, 4, 8, and 24 hours of ozone exposure are shown. The data were measured for the whole BBOA sample. The oxidation conditions were ozone concentration of 9 ppm, *T* = 293 K, and RH = 85%.

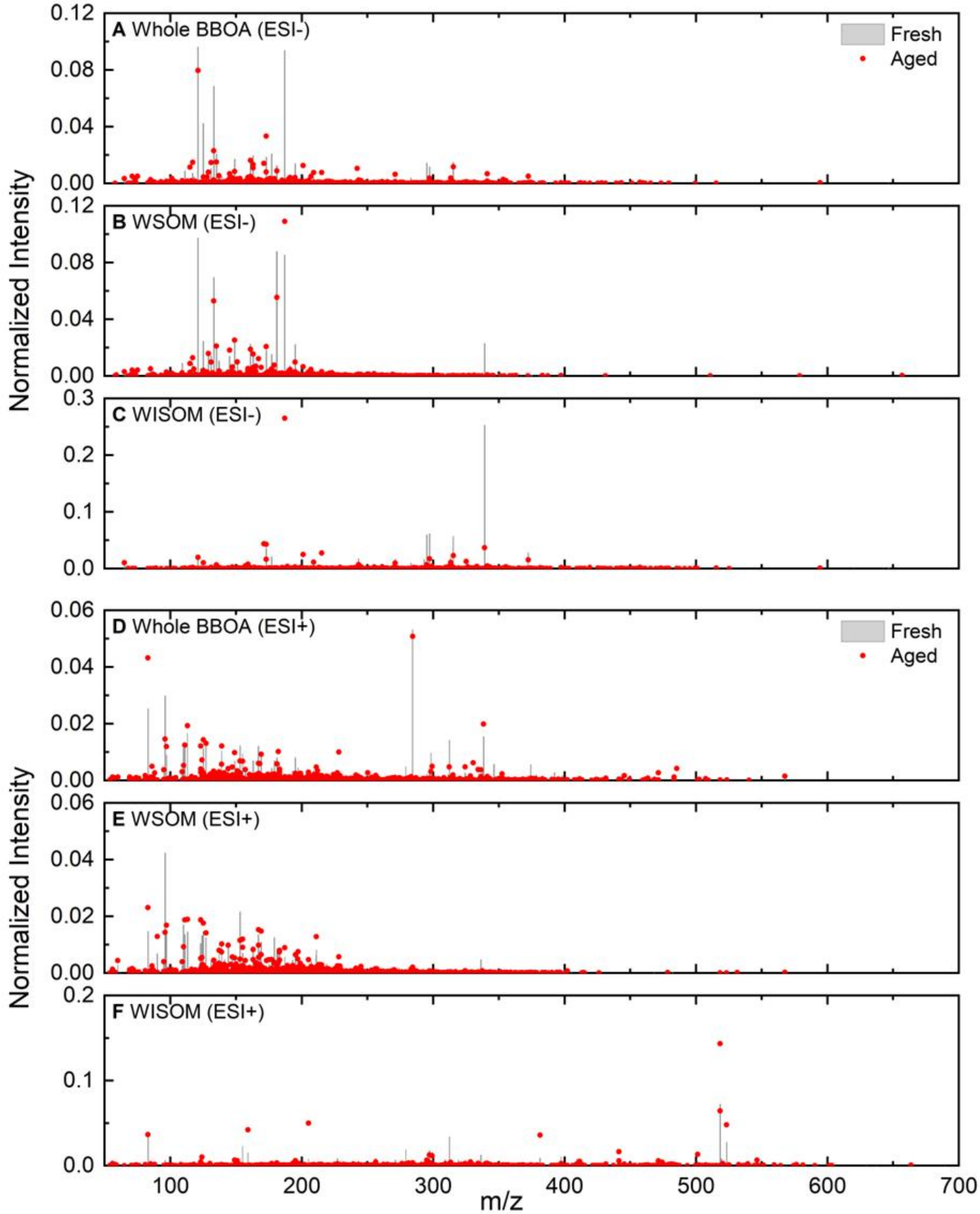

Fig. S4. ESI-MS data for BBOA samples. Both the negative (A: whole, B: WSOM, C: WISOM) and positive (D: whole, E: WSOM, F: WISOM) modes data are shown. The data for the fresh and aged samples are shown in grey bars and red dots, respectively. The oxidation conditions for the chemical aging were ozone exposure at 9 ppm for 8 (whole), 4 (WSOM) and 24 (WISOM) hours, T = 298 K (Whole) and 293 K (WSOM and WISOM), and RH = 85%.

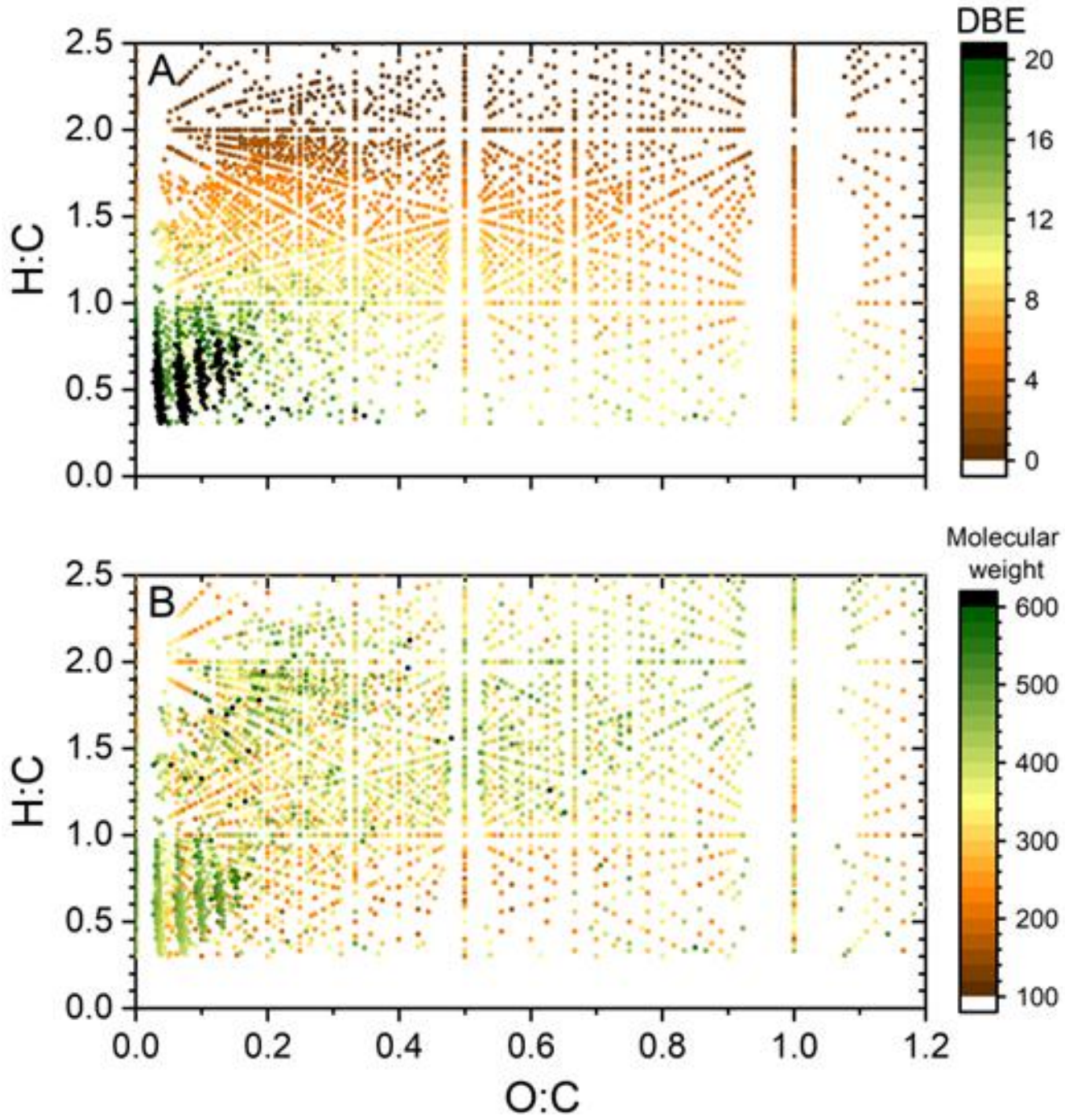

Fig. S5. Van Krevelen diagrams of compounds in the whole BBOA sample (fresh, ESI negative mode). The figure is colored by (A) DBE, and (B) molecular weight.

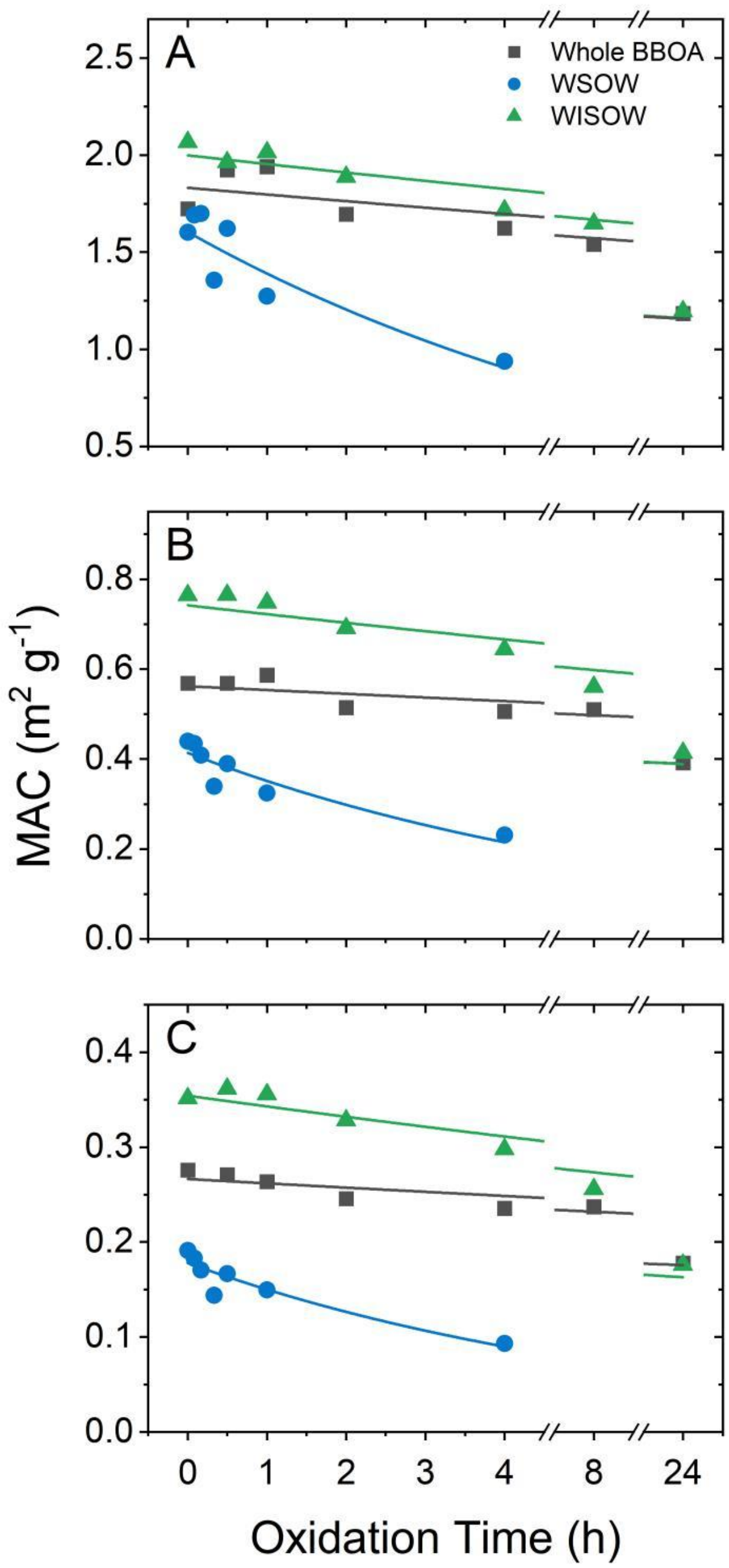

Fig. S6. Change of MAC by exposure to ozone. Data are shown for the whole, WSOM, and WISOM fractions of BBOA at wavelengths of (A) 300, (B) 360, and (C) 400 nm. The oxidation conditions for the chemical aging were ozone concentration of 9 ppm, T = 293 K, and RH = 85%. (B) is the same as Fig. 3A.

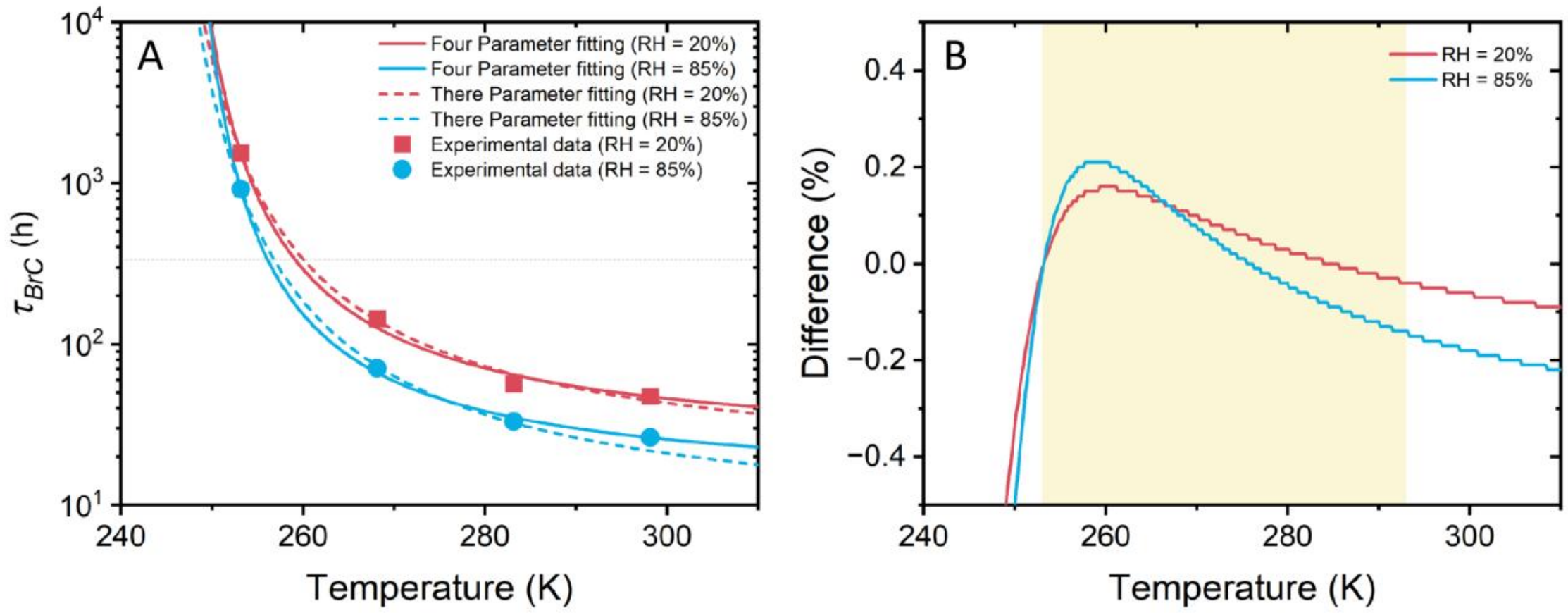

Fig. S7. Sensitivity of BrC bleaching on data fitting approaches. Comparisons between the three- and four- parameters fittings. (a) fitting results and (b) the differences between the two methods are shown. The yellow area in panel (b) is the experimental temperature range.

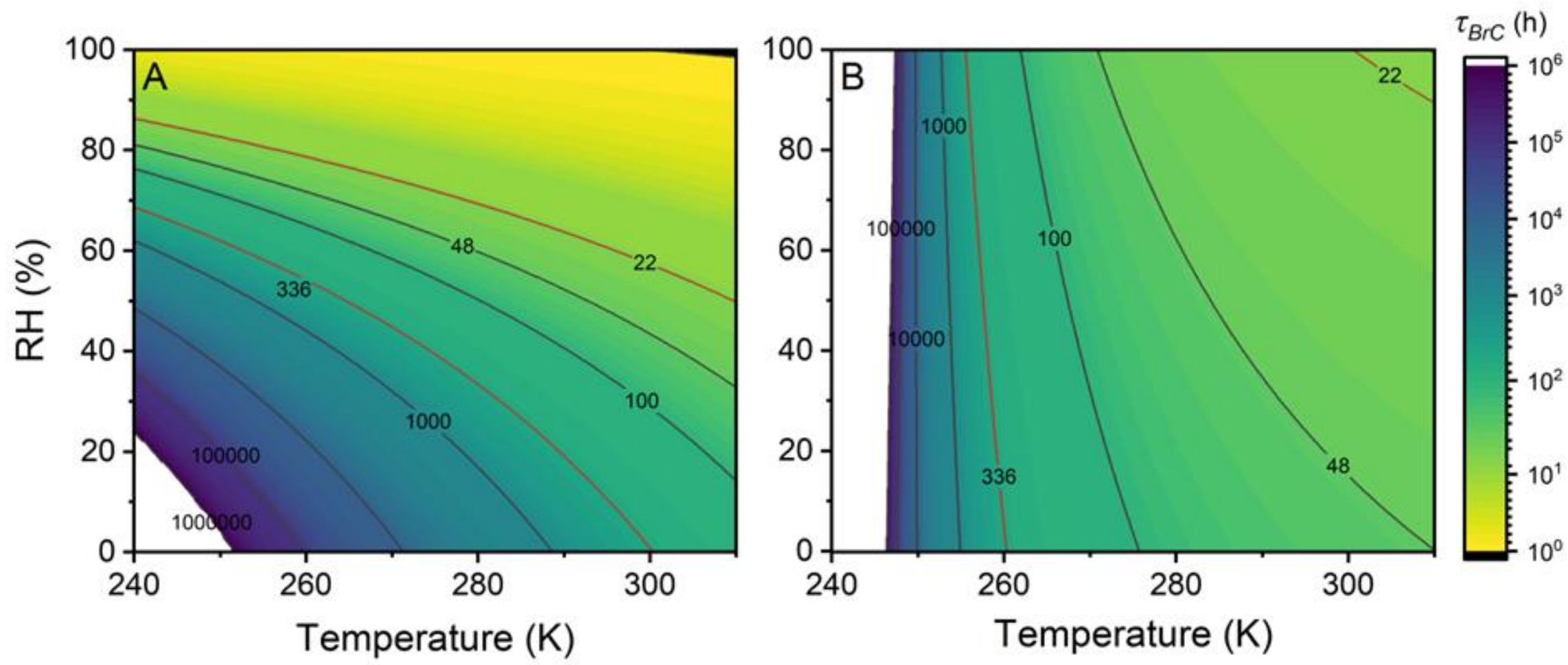

Fig. S8. Estimated bleaching lifetime of BrC as a function of temperature and RH. Comparison of τ_{BrC} by Schnitzler *et al.* (*31*) (left) and the present study (right). (B) is the same as Fig. 3D.

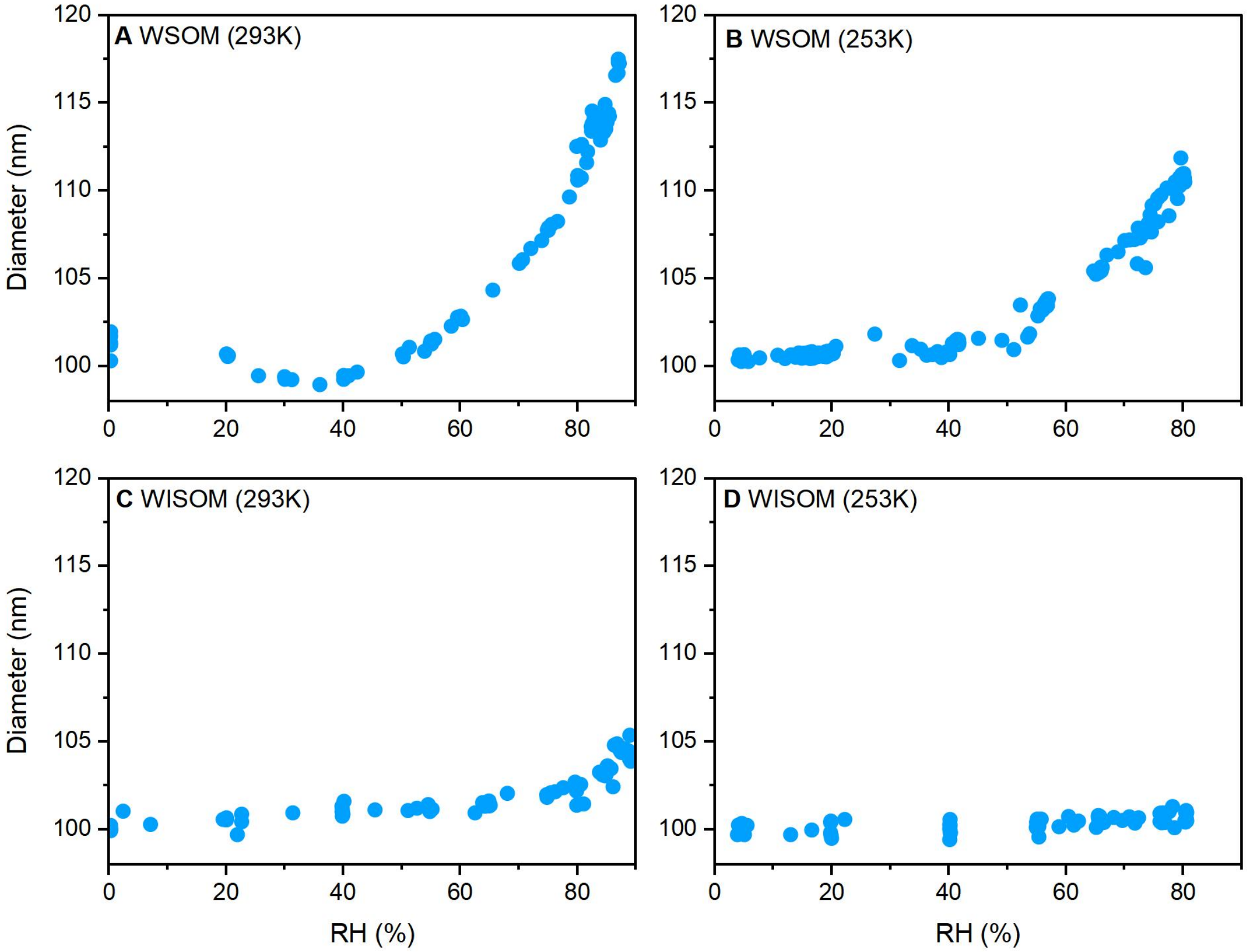

Fig. S9. Humidograms of the WSOM and WISOM samples. The results for the (A, B) WSOM and (C, D) WISOM samples at (A, C) 293K and (B, D) 253K are shown.

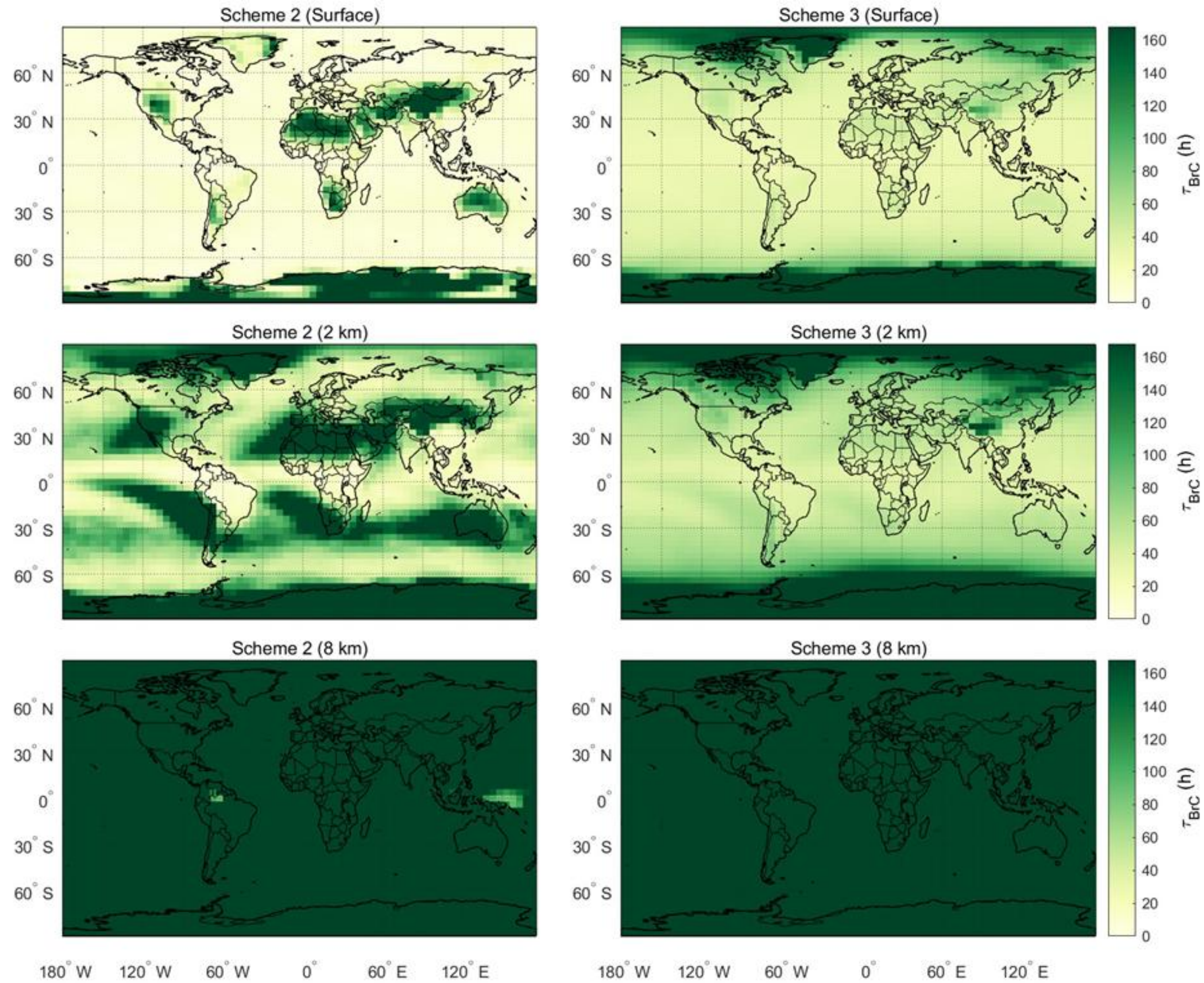

Fig. S10. Estimated globally bleaching lifetime of BrC. Comparison of τ_{BrC} by Schnitzler *et al.* (*31*) (Scheme 2, left panels) and the present study (Scheme 3, right panels) at (top) 0, (middle) 2, and (bottom) 8 km above the ground surface.

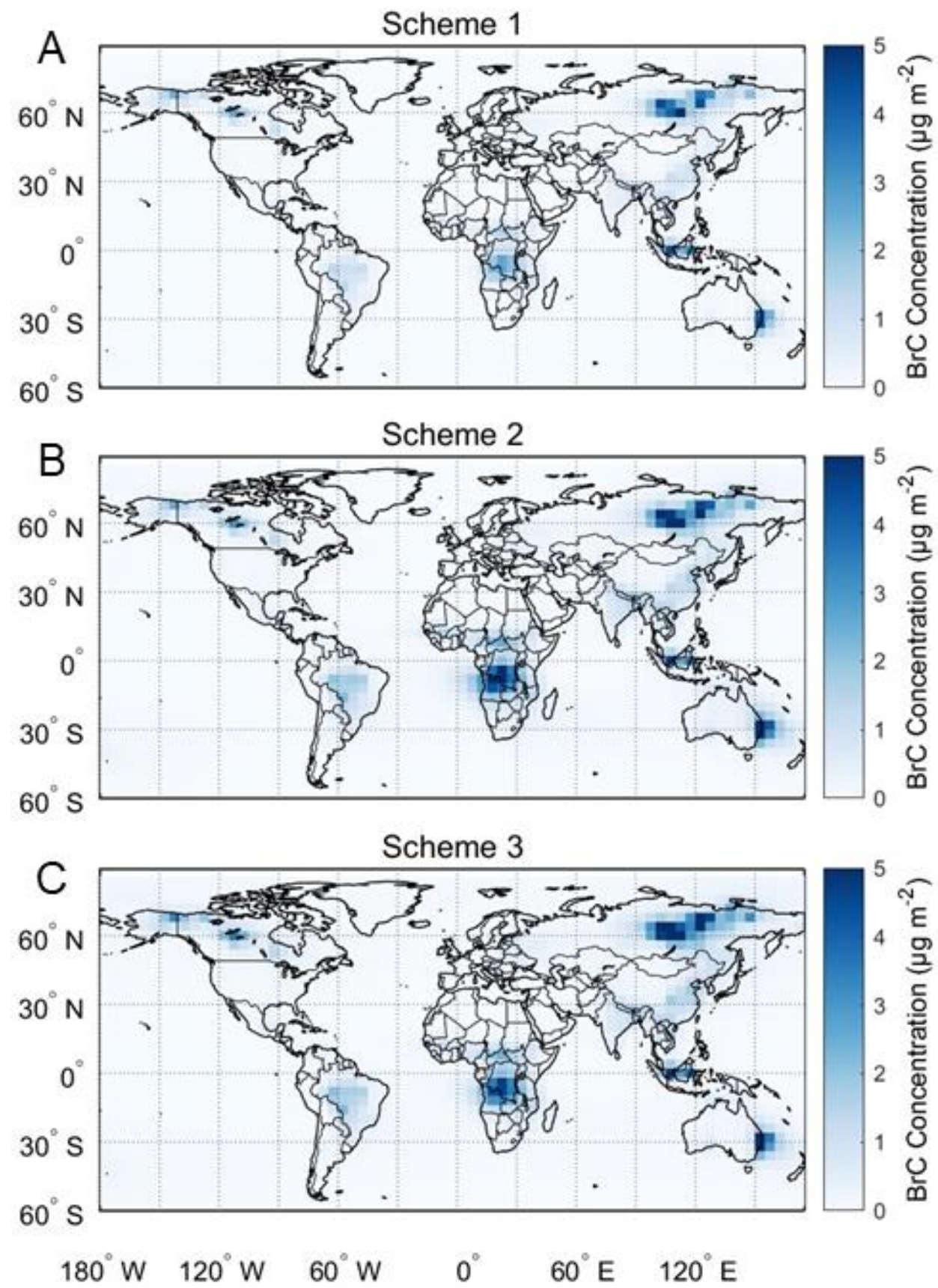

Fig. S11. Simulated fresh BrC column concentrations from biomass/biofuel combustions. Data are calculated using (A) default setting (Scheme 1), and parameterizations by (B) Schnitzler *et al.* (*31*) (Scheme 2) and (C) present study (Scheme 3). (C) is the same as Fig. 4A.

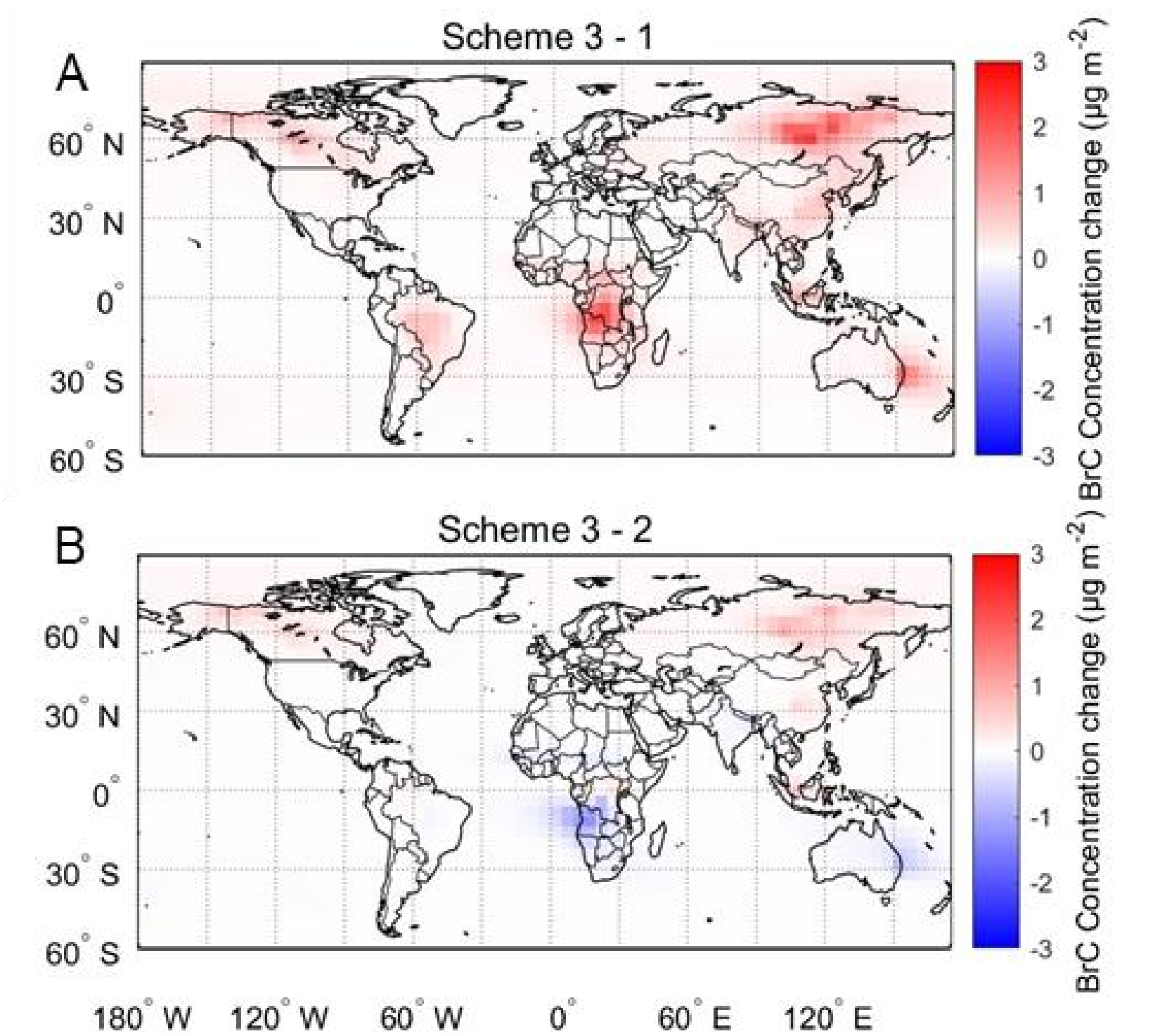

Fig. S12. Differences in simulated fresh BrC column concentrations from biomass/biofuel combustions. Data are calculated using (A) default setting (Scheme 1), and parameterization by (B) Schnitzler *et al.* (*31*) (Scheme 2) with the present study (Scheme 3).

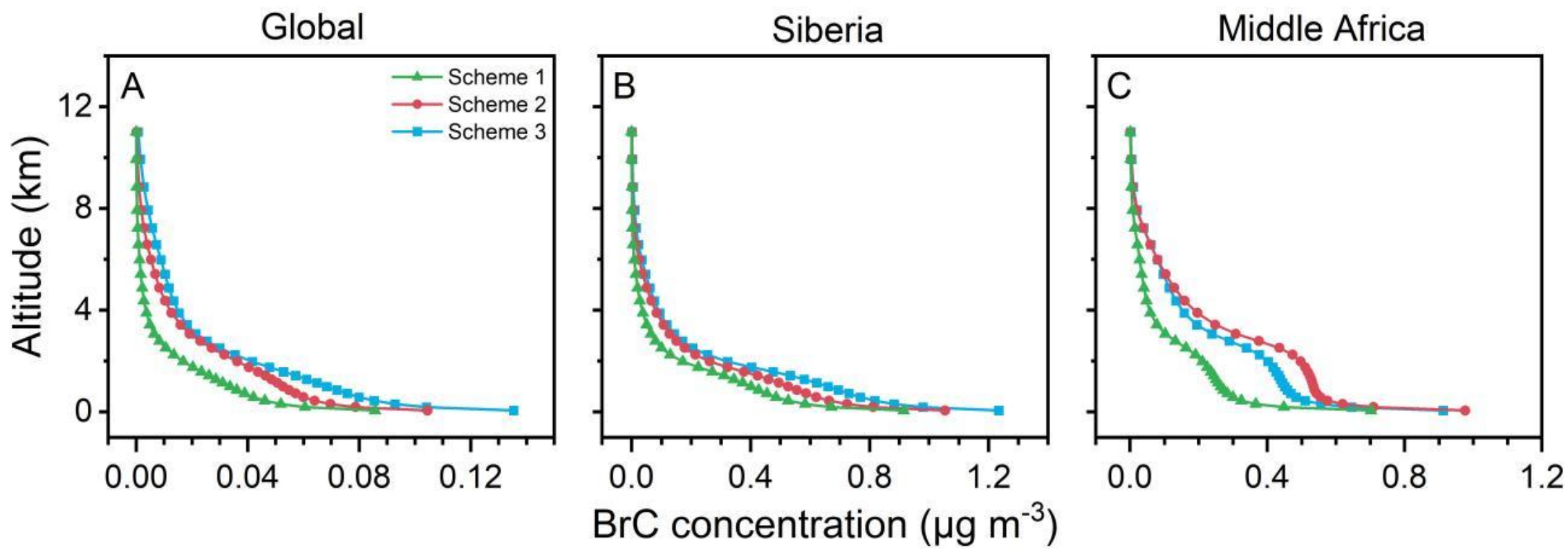

Fig. S13. Vertical profiles of simulated fresh BrC concentrations from biomass/biofuel combustions. Data are calculated using parameterizations by default setting (Scheme 1), Schnitzler *et al.* (*31*) (Scheme 2) and the present study (Scheme 3). The data for the (A) global average, (B) Siberia, and (C) middle Africa are shown. (A) is the same as Fig. 4B.

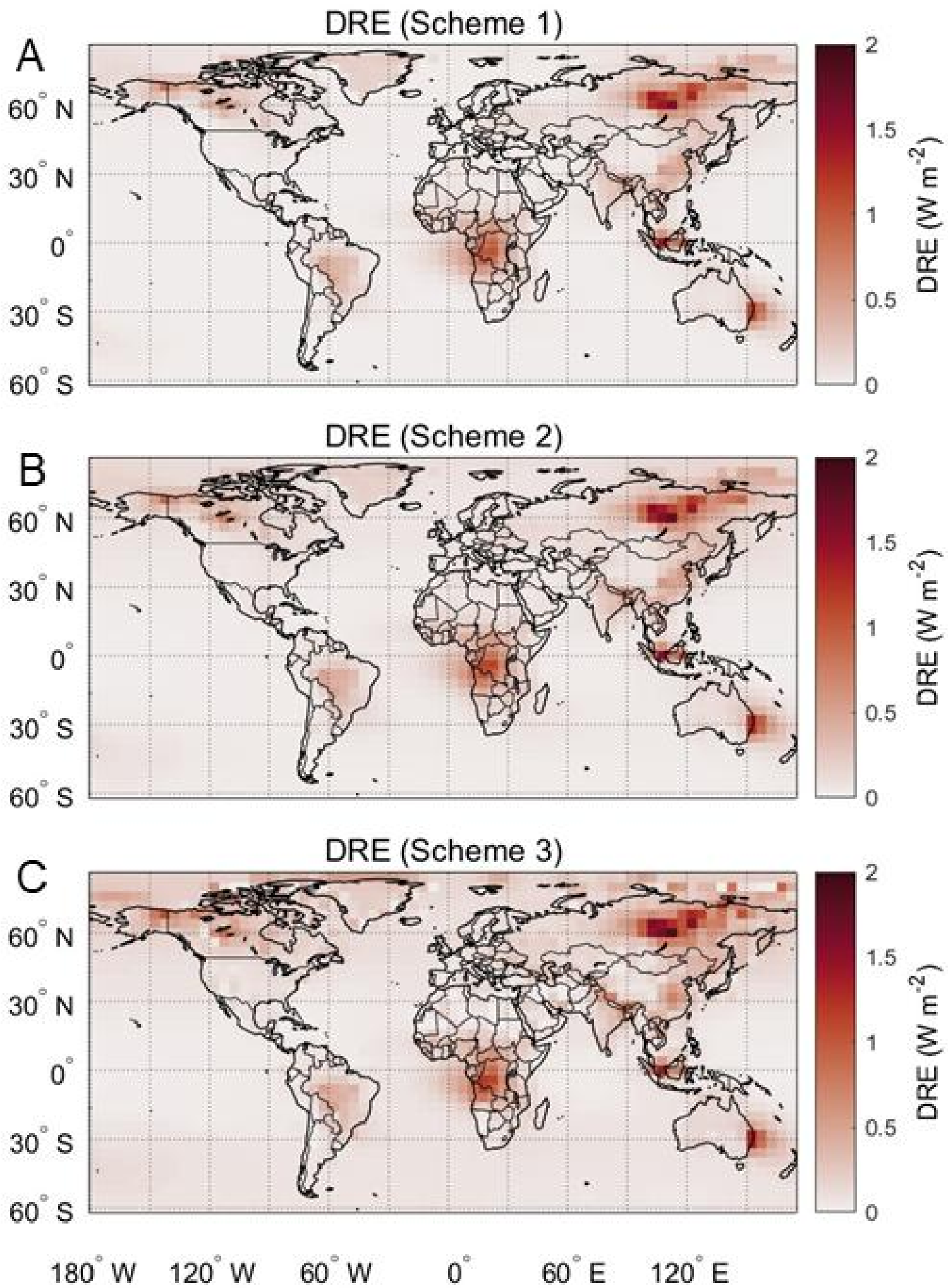

Fig. S14. Modeled DRE. Data are calculated using (A) default setting (Scheme 1), and parameterizations by (B) Schnitzler *et al.* (*31*) (Scheme 2) and (C) present study (Scheme 3).

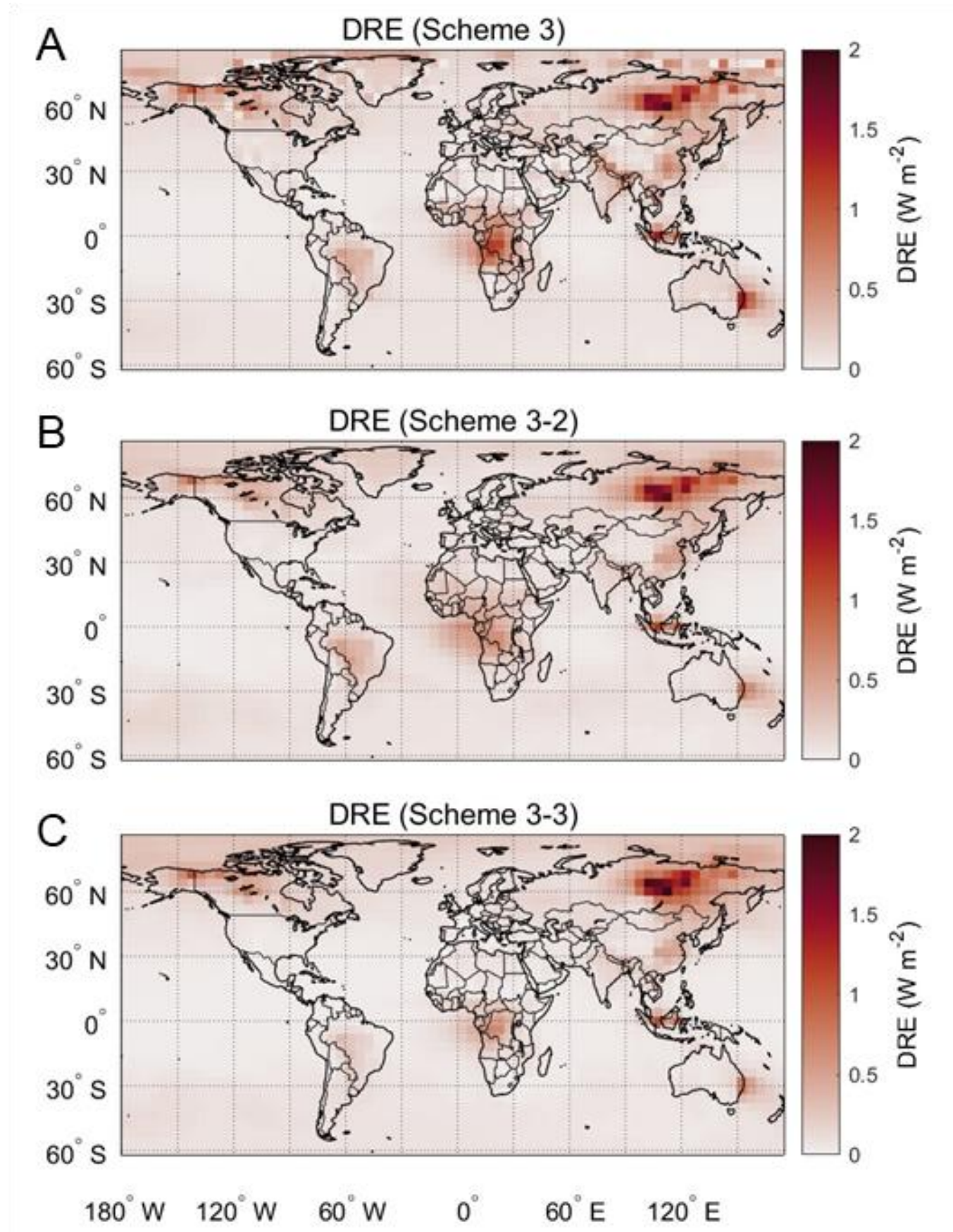

Fig. S15. Sensitivity of DRE on the assumed emission height. The results are calculated using the parameterization developed by the present study (scheme 3). Emission fractions under the PBL were assumed to be (A) 65%, (B) 0%, and (C) 100%.

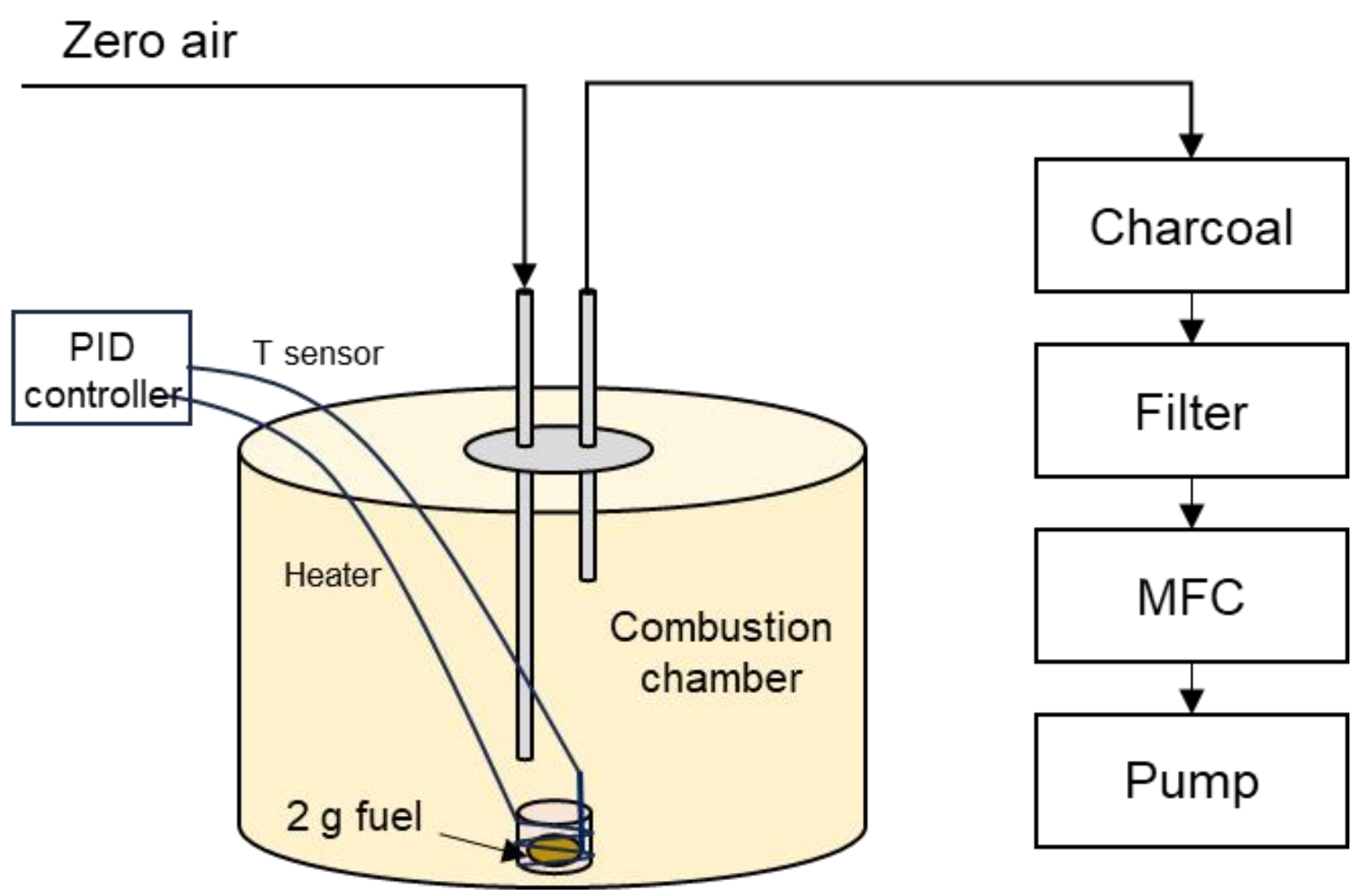

Fig. S16. Combustion setup for biomass burning.

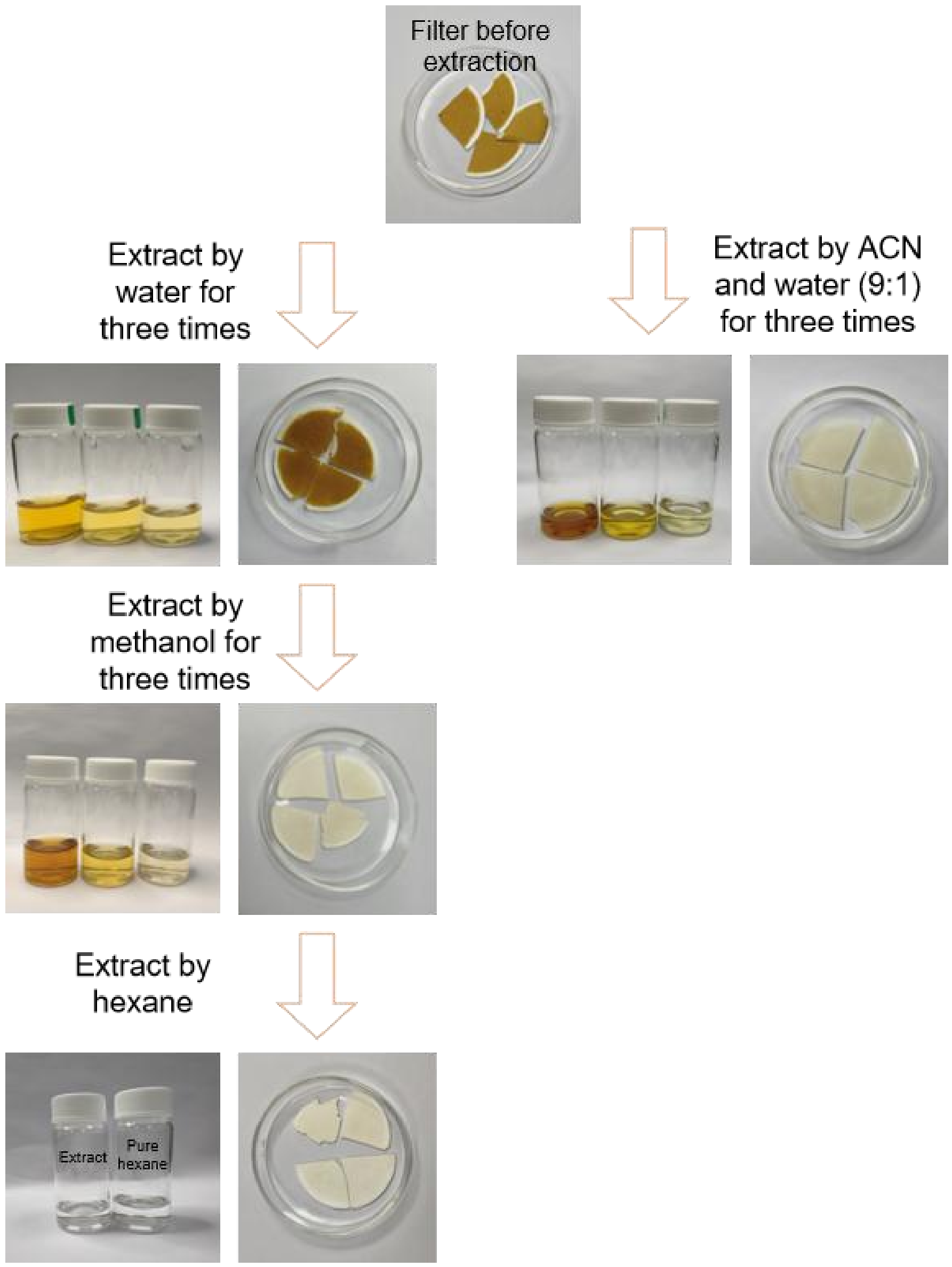

Fig. S17. Photographs of the BBOA extracts and the corresponding filter samples.

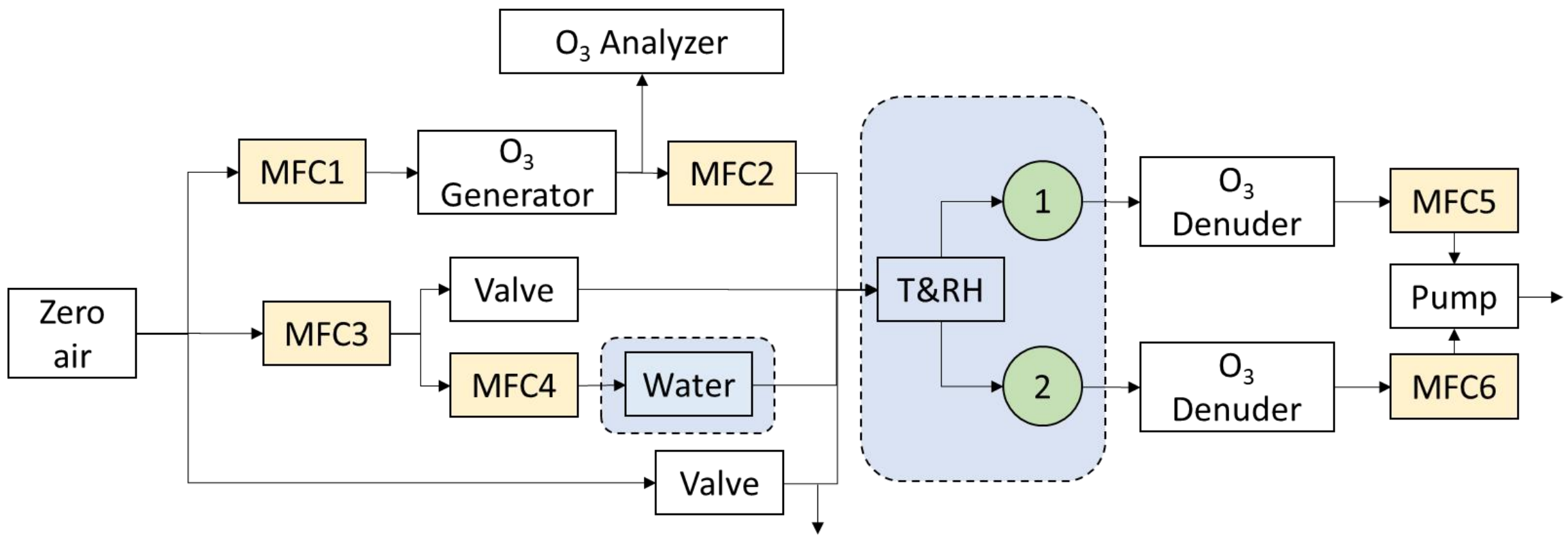

Fig. S18. Experimental setups for ozone exposure. Two filter samples (1 and 2 in the figure) were simultaneously oxidized under the same conditions. The areas colored by blue indicate that the temperature of these regions were precisely controlled using liquid circulators.

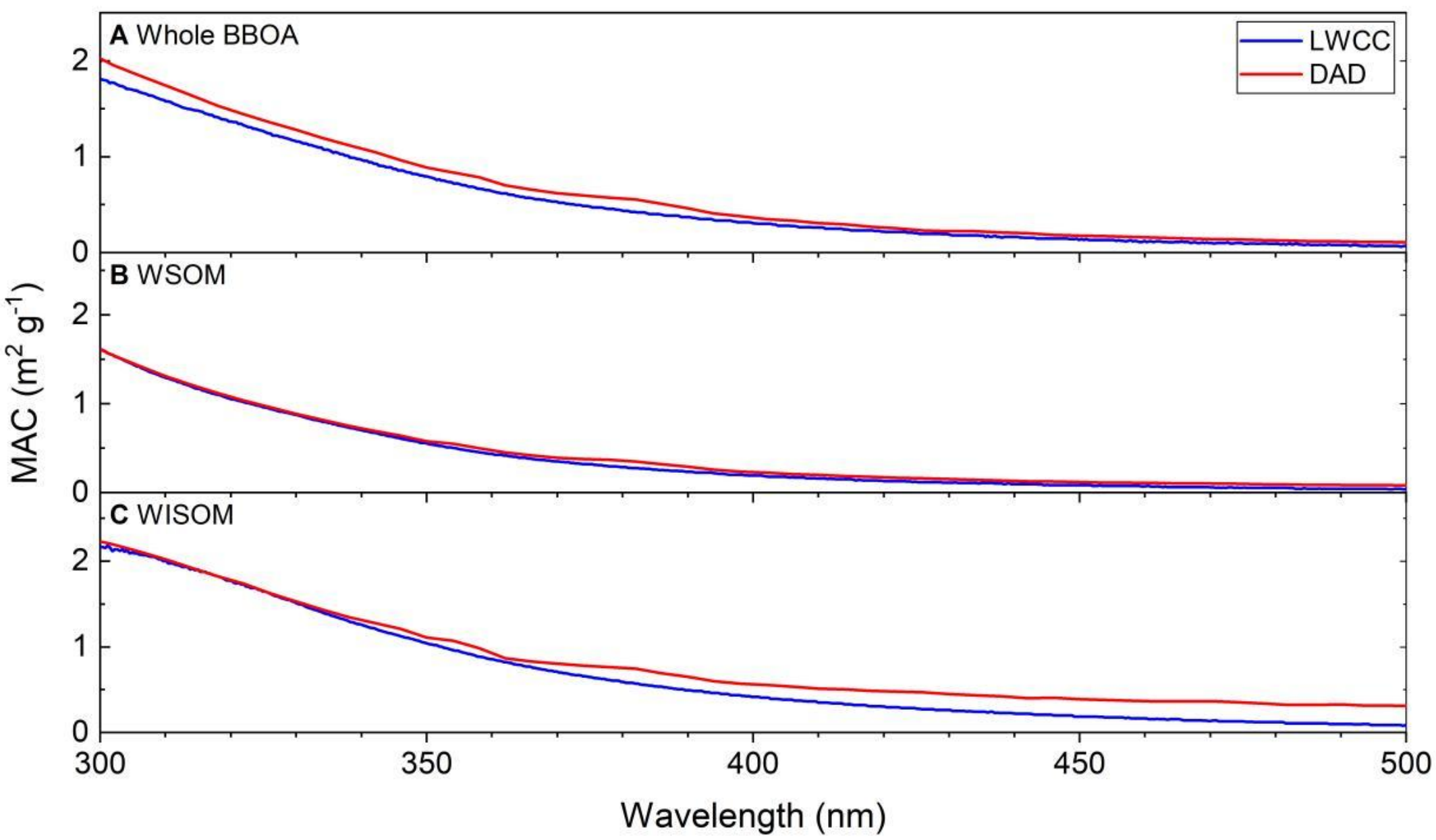

Fig. S19. Comparison of MAC measured by LWCC and DAD. Data for (A) whole, (B) WSOM, and (C) WISOM fractions of BBOA are shown.

Tables

Table S1. Summary of the UHLPC-HRMS data for fresh (-F) and aged (-A) samples. The data for both negative and positive modes of ESI are shown.

Mode	Sample	Average Formula	Molecular weight	Species number	O:C	H:C	N:C	DBE	T_g (K)
ESI-	Whole BBOA-F	$C_{9.2}H_{13.3}O_{3.5}N_{0.26}S_{0.07}$	186.05	2206	0.383	1.444	0.0235	3.693	254.6
	Whole BBOA-A	$C_{9}H_{13.4}O_{3.9}N_{0.35}S_{0.09}$	192.31	2269	0.438	1.486	0.0265	3.488	261.2
	WSOM-F	$C_{8.3}H_{11.3}O_{3.6}N_{0.18}S_{0.04}$	172.66	2691	0.433	1.361	0.0199	3.747	252.0
	WSOM-A	$C_{7.9}H_{10.5}O_{3.8}N_{0.23}S_{0.04}$	169.44	2593	0.480	1.334	0.0206	3.725	252.9
	WISOM-F	$C_{17.0}H_{26.8}O_{3.1}N_{0.29}S_{0.1}$	288.66	1243	0.184	1.576	0.0210	4.754	284.7
	WISOM-A	$C_{11.9}H_{19.7}O_{3.8}N_{0.26}S_{0.11}$	229.89	2102	0.318	1.656	0.0211	3.177	272.7
ESI+	Whole BBOA-F	$C_{11.5}H_{18.1}O_{1.7}N_{1.45}S_{0.11}$	206.95	4613	0.150	1.574	0.1664	4.168	223.3
	Whole BBOA-A	$C_{11.0}H_{17.6}O_{1.9}N_{1.49}S_{0.12}$	205.68	3880	0.176	1.593	0.1662	3.986	227.9
	WSOM-F	$C_{9.1}H_{12.8}O_{1.7}N_{1.48}S_{0.12}$	172.58	4810	0.181	1.415	0.1660	4.395	204.5
	WSOM-A	$C_{9.0}H_{12.9}O_{1.8}N_{1.47}S_{0.15}$	175.15	4804	0.199	1.436	0.1683	4.279	209.6
	WISOM-F	$C_{16.1}H_{27.5}O_{2.4}N_{1.4}S_{0.21}$	285.39	3538	0.152	1.714	0.1610	3.999	269.0
	WISOM-A	$C_{17.6}H_{31.2}O_{3.8}N_{1.4}S_{0.22}$	328.90	4134	0.216	1.780	0.1598	3.633	298.78

Table S2. Literature data for τ_{BrC} from field and laboratory studies. WS indicates 'water-soluble.'

Method	Reference	Bleaching lifetime (h)	BrC source	Oxidant	BrC fraction
Field observations	Forrister *et al.* (*46*)	13 - 21.5 *	US, SEAC4RS (Aug-Sep, 2013）		
	Chen *et al.* (*21*)	~23	North America (July-Aug,2013 - 2023)		
	Wong *et al.* (*24*)	15 - 18	Eastern Europe (July-Oct, 2016 - 2017)		
	Dasari *et al.* (19)	>100	South Asia (Jan - Mar, 2016)		WS
	Fang *et al.* (*25*)	67 - 184	East Asia (Jan, 2014)		WS
Laboratory experiments	Schnitzler *et al.* (*31*)	10 - 1000	Pine wood BOA	O_3	WS
	Wong *et al.* (*24*)	1 - 68	hardwood BOA	UVB/OH	WS
	O'Brien *et al.* (*22*)	~20	FIREX-AQ	Photolysis	
	Fleming *et al.* (*20*)	10 - 40 for typical BrC chromophores >100 for whole filter sample	FIREX	UV	
	Zhao *et al.* (*72*)	0.12 - 2.36 †	Imine BrC arising in the (GLYAS) (MGAS) solutions	UB/OH	
	Wang *et al.* (*73*)	1 - 17	BrC chromophores	OH	
	This study	26 - 1540	Straw BOA	O_3	

* Calculated from the half-life time of 9 - 15 h reported in the literature.
† Calculated from the half-life time of 5 - 98 min reported in the literature.

Table S3 Comparison of the experimental and parameterized values of τ_{BrC}.

RH (%)	Temperature (K)	τ_{BrC} (h)	
		Experiment	Parameterized
20	298.15	47.4	47.3
20	283.15	56.7	64.5
20	268.15	144.2	126.3
20	253.15	1542.1	1544.1
85	298.15	26.3	26.3
85	283.15	33.1	35.0
85	268.15	70.8	66.4
85	253.15	921.5	921.7
55	283.15	48.5	46.8

Table S4. Optimized parameters for the two parameterization methods at RH = 20% and 85%.

Equation	RH	A	B	T_0	C
S1-4	20%	21.132	95.414	242.154	0.0005197
	85%	20.617	83.340	243.506	0.0004085
S1-9	20%	20.3944	132.073		0.000544
	85%	19.709	139.603		0.000351

Table S5. List of all the experimental conditions.

Sample Type	Sample mass (mg)	Oxidation Condition		
		Time (h)	Temperature (K)	RH (%)
Whole BBOA	1.113	0		
	0.886	0		
	0.937	1	293.15	85
	1.059	4	293.15	85
	0.84	8	293.15	85
	1.383	16	293.15	85
	1.012	24	293.15	85
Whole BBOA	4.372	0		
	2.144	0		
	1.935	0.5	293.15	85
	2.001	1	293.15	85
	2.579	2	293.15	85
	3.258	4	293.15	85
	3.133	8	293.15	85
	3.456	24	293.15	85
WSOM	3.408	0		
	2.621	0		
	2.631	0.08	293.15	85
	2.863	0.17	293.15	85
	2.356	0.33	293.15	85
	2.919	0.5	293.15	85
	2.215	1	293.15	85
	2.489	4	293.15	85
WISOM	0.761	0		
	0.982	0		
	0.965	0.5	293.15	85
	0.98	1	293.15	85
	0.919	2	293.15	85
	1.038	4	293.15	85
	1.005	8	293.15	85
	1.223	24	293.15	85
Whole BBOA	1.087	0		
	0.777	0		
	0.939	8	298.15	85
	0.801	8	298.15	55
	0.971	8	298.15	20
	0.790	1	298.15	85
	0.813	1	298.15	55
	0.871	1	298.15	20
	1.426	8	283.15	85
	1.494	8	283.15	55
	1.576	8	283.15	20
	1.137	1	283.15	85

	1.095	1	283.15	55
	1.289	1	283.15	20
	0.706	8	268.15	85
	0.675	8	268.15	20
	0.710	8	253.15	85
	0.736	8	253.15	20
	0.690	1	253.15	85
	0.748	1	253.15	20
Filter Blank	66<br>58<br>43<br>44<br>33<br>42	2	293.15	85

Table S6. Sensitivity of the emission fraction above PBL on the global average DRE.

Scheme	τ_{BrC} (h)	Emission fraction above PBL	DRE (W m^{-2})
**Scheme 3**	parameterization by the present study (whole BBOA particles)	35%	0.125
**Scheme 3-2**		0%	0.117
**Scheme 3-3**		100%	0.093

Table S7. Sampling conditions for BBOA.

Filter type	Sample flow volume (L)	Sample flow rate (slpm)	Collection time (min)	Sample usage
PTFE	1.5	2	0.75	Change in oxidation T and RH
PTFE	1.5	1.5	1	Change in oxidation time
PTFE	6	3	2	Change in oxidation time
Glass fiber	90 ~ 120	3	30 ~ 40	Extraction by water and methanol

REFERENCES

1. IPCC, *Climate Change 2023: Synthesis Report. Contribution of Working Groups I, II and III to the Sixth Assessment Report of the Intergovernmental Panel on Climate Change*, H. Lee, J. Romero, Eds. (IPCC, 2023).

2. J. Li, B. E. Carlson, Y. L. Yung, D. Lv, J. Hansen, J. E. Penner, H. Liao, V. Ramaswamy, R. A. Kahn, P. Zhang, O. Dubovik, A. Ding, A. A. Lacis, L. Zhang, Y. Dong, Scattering and absorbing aerosols in the climate system. *Nat. Rev. Earth Environ.* **3**, 363–379 (2022).

3. J. L. Jimenez, M. R. Canagaratna, N. M. Donahue, A. S. H. Prevot, Q. Zhang, J. H. Kroll, P. F. DeCarlo, J. D. Allan, H. Coe, N. L. Ng, A. C. Aiken, K. S. Docherty, I. M. Ulbrich, A. P. Grieshop, A. L. Robinson, J. Duplissy, J. D. Smith, K. R. Wilson, V. A. Lanz, C. Hueglin, Y. L. Sun, J. Tian, A. Laaksonen, T. Raatikainen, J. Rautiainen, P. Vaattovaara, M. Ehn, M. Kulmala, J. M. Tomlinson, D. R. Collins, M. J. Cubison, E. J. Dunlea, J. A. Huffman, T. B. Onasch, M. R. Alfarra, P. I. Williams, K. Bower, Y. Kondo, J. Schneider, F. Drewnick, S. Borrmann, S. Weimer, K. Demerjian, D. Salcedo, L. Cottrell, R. Griffin, A. Takami, T. Miyoshi, S. Hatakeyama, A. Shimono, J. Y. Sun, Y. M. Zhang, K. Dzepina, J. R. Kimmel, D. Sueper, J. T. Jayne, S. C. Herndon, A. M. Trimborn, L. R. Williams, E. C. Wood, A. M. Middlebrook, C. E. Kolb, U. Baltensperger, D. R. Worsnop, Evolution of organic aerosols in the atmosphere. *Science* **326**, 1525–1529 (2009).

4. Q. Zhang, J. L. Jimenez, M. R. Canagaratna, J. D. Allan, H. Coe, I. Ulbrich, M. R. Alfarra, A. Takami, A. M. Middlebrook, Y. L. Sun, K. Dzepina, E. Dunlea, K. Docherty, P. F. DeCarlo, D. Salcedo, T. Onasch, J. T. Jayne, T. Miyoshi, A. Shimono, S. Hatakeyama, N. Takegawa, Y. Kondo, J. Schneider, F. Drewnick, S. Borrmann, S. Weimer, K. Demerjian, P. Williams, K. Bower, R. Bahreini, L. Cottrell, R. J. Griffin, J. Rautiainen, J. Y. Sun, Y. M. Zhang, D. R. Worsnop, Ubiquity and dominance of oxygenated species in organic aerosols in anthropogenically-influenced Northern Hemisphere midlatitudes. *Geophys. Res. Lett.* **34**, L13801 (2007).

5. T. Moise, J. M. Flores, Y. Rudich, Optical properties of secondary organic aerosols and their changes by chemical processes. *Chem. Rev.* **115**, 4400–4439 (2015).

6. A. Laskin, J. Laskin, S. A. Nizkorodov, Chemistry of atmospheric brown carbon. *Chem. Rev.* **115**, 4335–4382 (2015).

7. R. Saleh, From measurements to models: Toward accurate representation of brown carbon in climate calculations. *Curr. Pollut. Rep.* **6**, 90–104 (2020).

8. A. Zhang, Y. Wang, Y. Zhang, R. J. Weber, Y. Song, Z. Ke, Y. Zou, Modeling the global radiative effect of brown carbon: A potentially larger heating source in the tropical free troposphere than black carbon. *Atmos. Chem. Phys.* **20**, 1901–1920 (2020).

9. A. Laskin, C. P. West, A. P. S. Hettiyadura, Molecular insights into the composition, sources, and aging of atmospheric brown carbon. *Chem. Soc. Rev.* **54**, 1583–1612 (2025).

10. D. S. Jo, R. J. Park, S. Lee, S. W. Kim, X. Zhang, A global simulation of brown carbon: Implications for photochemistry and direct radiative effect. *Atmos. Chem. Phys.* **16**, 3413–3432 (2016).

11. Y. Iinuma, E. Brüggemann, T. Gnauk, K. Müller, M. O. Andreae, G. Helas, R. Parmar, H. Herrmann, Source characterization of biomass burning particles: The combustion of selected European conifers, African hardwood, savanna grass, and German and Indonesian peat.*J. Geophys. Res. Atmos.* **112**, 209 (2007).

12. T. Cao, M. Li, C. Zou, X. Fan, J. Song, W. Jia, C. Yu, Z. Yu, P. Peng, Chemical composition, optical properties, and oxidative potential of water- and methanol-soluble organic compounds emitted from the combustion of biomass materials and coal. *Atmos. Chem. Phys.* **21**, 13187–13205 (2021).

13. X. Jiang, D. Liu, Q. Li, P. Tian, Y. Wu, S. Li, K. Hu, S. Ding, K. Bi, R. Li, M. Huang, D. Ding, Q. Chen, S. Kong, W. Li, Y. Pang, D. He, Connecting the light absorption of atmospheric organic aerosols with oxidation state and polarity. *Environ. Sci. Technol.* **56**, 12873–12885 (2022).

14. Q. Chen, F. Ikemori, Y. Nakamura, P. Vodicka, K. Kawamura, M. Mochida, Structural and light-absorption characteristics of complex water-insoluble organic mixtures in urban submicrometer aerosols. *Environ. Sci. Technol.* **51**, 8293–8303 (2017).

15. S. Afsana, R. Zhou, Y. Miyazaki, E. Tachibana, D. K. Deshmukh, K. Kawamura, M. Mochida, Abundance, chemical structure, and light absorption properties of humic-like substances (HULIS) and other organic fractions of forest aerosols in Hokkaido. *Sci. Rep.* **12**, 14379 (2022).

16. R.-J. Huang, L. Yang, J. Shen, W. Yuan, Y. Gong, J. Guo, W. Cao, J. Duan, H. Ni, C. Zhu, W. Dai, Y. Li, Y. Chen, Q. Chen, Y. Wu, R. Zhang, U. Dusek, C. O'Dowd, T. Hoffmann, Water-insoluble organics dominate brown carbon in wintertime urban aerosol of China: Chemical characteristics and optical properties. *Environ. Sci. Technol.* **54**, 7836–7847 (2020).

17. K. Bali, S. Banerji, J. R. Campbell, A. V. Bhakta, L. W. A. Chen, C. D. Holmes, J. Mao, Measurements of brown carbon and its optical properties from boreal forest fires in Alaska summer. *Atmos. Environ.* **324**, 120436 (2024).

18. C. K. Glenn, O. El Hajj, Z. McQueen, R. P. Poland, R. Penland, E. T. Roberts, J. H. Choi, B. Bai, N. Shin, A. Anosike, K. V. Kumar, M. I. Abdurrahman, P. Liu, I. J. Amster, G. D. Smith, S. Flanagan, M. A. Callaham, E. L. Loudermilk, J. J. O'Brien, R. Saleh, Brown carbon emissions from biomass burning under simulated wildfire and prescribed-fire conditions. *ACS EST Air* **1**, 1124–1136 (2024).

19. S. Dasari, A. Andersson, S. Bikkina, H. Holmstrand, K. Budhavant, S. Satheesh, E. Asmi, J. Kesti, J. Backman, A. Salam, D. S. Bisht, S. Tiwari, Z. Hameed, Ö. Gustafsson, Photochemical degradation affects the light absorption of water-soluble brown carbon in the South Asian outflow. *Sci. Adv.* **5**, eaau8066 (2019).

20. L. T. Fleming, P. Lin, J. M. Roberts, V. Selimovic, R. Yokelson, J. Laskin, A. Laskin, S. A. Nizkorodov, Molecular composition and photochemical lifetimes of brown carbon chromophores in biomass burning organic aerosol. *Atmos. Chem. Phys.* **20**, 1105–1129 (2020).

21. J.-H. Chen, U. Puttu, H. N. Huynh, A. T. Ahern, K. Ball, K. H. Bates, C. A. Brock, T. Campos, M. M. Coggon, J. D. Crounse, J. de Gouw, J. P. DiGangi, G. S. Diskin, G. I. Gkatzelis, H. S. Halliday, L. Hu, A. R. Koss, Y. Li, M. Lyu, G. Michailoudi, S. M. Murphy, J. B. Nowak, B. B. Palm, J. Peischl, W. Permar, A. E. Perring, R. P. Pokhrel, N. B. Schafer, J. P. Schwarz, K. Sekimoto, V. Selimovic, C. E. Stockwell, A. P. Sullivan, J. A. Thornton, N. L. Wagner, S. Wang, C. Warneke, P. O. Wennberg, L. Zeng, R. J. Yokelson, R. J. Weber, L. Xu, Atmospheric evolution of brown carbon from wildfires in North America. *Environ. Sci. Technol.* **59**, 17145–17159 (2025).

22. R. O'Brien, H. Yu, N. Warren, C. Robinson, C. Y. Lim, C. E. Jordan, B. E. Anderson, J. H. Kroll, C. D. Cappa, Changes in light absorption and chemical properties for biomass burning organic aerosol over long time scales. *ACS EST Air* **2**, 773–785 (2025).

23. X. Wang, C. L. Heald, A. J. Sedlacek, S. S. de Sá, S. T. Martin, M. L. Alexander, T. B. Watson, A. C. Aiken, S. R. Springston, P. Artaxo, Deriving brown carbon from multiwavelength absorption measurements: Method and application to AERONET and Aethalometer observations. *Atmos. Chem. Phys.* **16**, 12733–12752 (2016).

24. J. P. S. Wong, M. Tsagkaraki, I. Tsiodra, N. Mihalopoulos, K. Violaki, M. Kanakidou, J. Sciare, A. Nenes, R. J. Weber, Atmospheric evolution of molecular-weight-separated brown carbon from biomass burning. *Atmos. Chem. Phys.* **19**, 7319–7334 (2019).

25. W. Fang, A. Andersson, M. Lee, M. Zheng, K. Du, S.-W. Kim, H. Holmstrand, Ö. Gustafsson, Combined influences of sources and atmospheric bleaching on light absorption of water-soluble brown carbon aerosols. *npj Clim. Atmos. Sci.* **6**, 104 (2023).

26. X. Wang, C. L. Heald, J. Liu, R. J. Weber, P. Campuzano-Jost, J. L. Jimenez, J. P. Schwarz, A. E. Perring, Exploring the observational constraints on the simulation of brown carbon. *Atmos. Chem. Phys.* **18**, 635–653 (2018).

27. Y. Li, T.-M. Fu, J. Z. Yu, A. Zhang, X. Yu, J. Ye, L. Zhu, H. Shen, C. Wang, X. Yang, S. Tao, Q. Chen, Y. Li, L. Li, H. Che, C. L. Heald, Nitrogen dominates global atmospheric organic aerosol absorption. *Science* **387**, 989–995 (2025).

28. M. Shiraiwa, M. Ammann, T. Koop, U. Poschl, Gas uptake and chemical aging of semisolid organic aerosol particles. *Proc. Natl. Acad. Sci. U.S.A.* **108**, 11003–11008 (2011).

29. Q. Mu, M. Shiraiwa, M. Octaviani, N. Ma, A. Ding, H. Su, G. Lammel, U. Pöschl, Y. Cheng, Temperature effect on phase state and reactivity controls atmospheric multiphase chemistry and transport of PAHs. *Sci. Adv.* **4**, eaap7314 (2018).

30. V. J. Baboomian, G. V. Crescenzo, Y. Huang, F. Mahrt, M. Shiraiwa, A. K. Bertram, S. A. Nizkorodov, Sunlight can convert atmospheric aerosols into a glassy solid state and modify their environmental impacts. *Proc. Natl. Acad. Sci. U.S.A.* **119**, e2208121119 (2022).

31. E. G. Schnitzler, N. G. A. Gerrebos, T. S. Carter, Y. Huang, C. L. Heald, A. K. Bertram, J. P. D. Abbatt, Rate of atmospheric brown carbon whitening governed by environmental conditions. *Proc. Natl. Acad. Sci. U.S.A.* **119**, e2205610119 (2022).

32. M. Kuwata, S. T. Martin, Phase of atmospheric secondary organic material affects its reactivity. *Proc. Natl. Acad. Sci. U.S.A.* **109**, 17354–17359 (2012).

33. J. Li, S. M. Forrester, D. A. Knopf, Heterogeneous oxidation of amorphous organic aerosol surrogates by O_3, NO_3, and OH at typical tropospheric temperatures. *Atmos. Chem. Phys.* **20**, 6055–6080 (2020).

34. W. Liu, L. He, Y. Liu, K. Liao, Q. Chen, M. Kuwata, Suppressed atmospheric chemical aging of cooking organic aerosol particles in wintertime conditions. *Atmos. Chem. Phys.* **24**, 5625–5636 (2024).

35. M. Cheng, Y. Li, M. Kuwata, Hysteresis in water content of ultrafine glassy organic aerosol particles. *J. Geophys. Res. Atmos.* **129**, e2024JD041440 (2024).

36. K. Siemens, T. Paik, A. Li, F. Rivera-Adorno, J. Tomlin, Q. Xie, R. K. Chakrabarty, A. Laskin, Light absorption and chemical composition of brown carbon organic aerosol produced from burning of selected biofuels. *ACS Earth Space Chem.* **8**, 1416–1428 (2024).

37. X. Xie, Y. Zhang, R. Liang, X. Wang, Global modeling of brown carbon: Impact of temperature- and humidity-dependent bleaching. *Atmos. Chem. Phys.* **25**, 13547–13561 (2025).

38. F. K. A. Gregson, N. G. A. Gerrebos, M. Schervish, S. Nikkho, E. G. Schnitzler, C. Schwartz, C. Carlsten, J. P. D. Abbatt, S. Kamal, M. Shiraiwa, A. K. Bertram, Phase behavior and viscosity in biomass burning organic aerosol and climatic impacts. *Environ. Sci. Technol.* **57**, 14548–14557 (2023).

39. R. Satish, N. Rastogi, A. Singh, D. Singh, Change in characteristics of water-soluble and water-insoluble brown carbon aerosols during a large-scale biomass burning. *Environ. Sci. Pollut. Res.* **27**, 33339–33350 (2020).

40. R. Zhou, Q. Chen, J. Chen, L. Ren, Y. Deng, P. Vodicka, D. K. Deshmukh, K. Kawamura, P. Fu, M. Mochida, Distinctive sources govern organic aerosol fractions with different degrees of oxygenation in the urban atmosphere. *Environ. Sci. Technol.* **55**, 4494–4503 (2021).

41. J. P. S. Wong, A. Nenes, R. J. Weber, Changes in light absorptivity of molecular weight separated brown carbon due to photolytic aging. *Environ. Sci. Technol.* **51**, 8414–8421 (2017).

42. T. He, Y. Wu, D. Wang, J. Cai, J. Z. Song, Z. Q. Yu, X. Y. Zeng, P. Peng, Molecular compositions and optical properties of water-soluble brown carbon during the autumn and winter in Guangzhou, China. *Atmos. Environ.* **296**, 119573 (2023).

43. N. Xu, M. Hu, X. Li, L. Zeng, Y. Wang, Y. Qiu, K. Song, S. Li, S. Shuai, Y. Chen, J. Hu, S. Guo, Unraveling the light-absorbing properties of brown carbon at a molecular level. *Geophys. Res. Lett.* **51**, e2024GL108834 (2024).

44. W. S. W. DeRieux, Y. Li, P. Lin, J. Laskin, A. Laskin, A. K. Bertram, S. A. Nizkorodov, M. Shiraiwa, Predicting the glass transition temperature and viscosity of secondary organic material using molecular composition. *Atmos. Chem. Phys.* **18**, 6331–6351 (2018).

45. T. Koop, J. Bookhold, M. Shiraiwa, U. Pöschl, Glass transition and phase state of organic compounds: Dependency on molecular properties and implications for secondary organic aerosols in the atmosphere. *Phys. Chem. Chem. Phys.* **13**, 19238–19255 (2011).

46. H. Forrister, J. Liu, E. Scheuer, J. Dibb, L. Ziemba, K. L. Thornhill, B. Anderson, G. Diskin, A. E. Perring, J. P. Schwarz, P. Campuzano-Jost, D. A. Day, B. B. Palm, J. L. Jimenez, A. Nenes, R. J. Weber, Evolution of brown carbon in wildfire plumes. *Geophys. Res. Lett.* **42**, 4623–4630 (2015).

47. D. R. Worsnop, J. W. Morris, Q. Shi, P. Davidovits, C. E. Kolb, A chemical kinetic model for reactive transformations of aerosol particles. *Geophys. Res. Lett.* **29**, 54–85 (2002).

48. B. Zobrist, V. Soonsin, B. P. Luo, U. K. Krieger, C. Marcolli, T. Peter, T. Koop, Ultra-slow water diffusion in aqueous sucrose glasses. *Phys. Chem. Chem. Phys.* **13**, 3514–3526 (2011).

49. R. Subramanian, C. A. Roden, P. Boparai, T. C. Bond, Yellow beads and missing particles: Trouble ahead for filter-based absorption measurements. *Aerosol Sci. Tech.* **41**, 630–637 (2007).

50. C. L. Heald, D. A. Ridley, J. H. Kroll, S. R. H. Barrett, K. E. Cady-Pereira, M. J. Alvarado, C. D. Holmes, Contrasting the direct radiative effect and direct radiative forcing of aerosols. *Atmos. Chem. Phys.* **14**, 5513–5527 (2014).

51. J. Duan, R. J. Huang, C. Lin, J. Qu, J. Liu, W. Huang, Y. Zhan, W. Yuan, T. Wang, L. Zhou, W. Xu, Q. Liu, Z. Liu, S. Lou, H. Yang, D. D. Huang, C. Huang, H. Wang, Relative humidity modulates photochemical aging of light-absorbing carbonaceous aerosols: Insights from ambient oxidation flow reactor. *Environ. Sci. Technol.* **59**, 24481–24491 (2025).

52. L. J. Li, Y. M. Han, J. J. Li, Y. Lin, X. Zhang, Q. Y. Wang, J. J. Cao, Effects of photochemical aging on the molecular composition of organic aerosols derived from agricultural biomass burning in whole combustion process. *Sci. Total Environ.* **946**, 174152 (2024).

53. S. H. Budisulistiorini, J. Chen, M. Itoh, M. Kuwata, Can online aerosol mass spectrometry analysis classify secondary organic aerosol (SOA) and oxidized primary organic aerosol (OPOA)? A case study of laboratory and field studies of Indonesian biomass burning. *ACS Earth Space Chem.* **5**, 3511–3522 (2021).

54. S. Bogler, J. Zhang, R. K. Y. Cheung, K. Li, A. S. H. Prévôt, I. El Haddad, D. M. Bell, Ozonolysis of primary biomass burning organic aerosol particles: Insights into reactivity and phase state. *Atmos. Chem. Phys.* **25**, 10229–10243 (2025).

55. J. K. Kodros, C. Kaltsonoudis, M. Paglione, K. Florou, S. Jorga, C. Vasilakopoulou, M. Cirtog, M. Cazaunau, B. Picquet-Varrault, A. Nenes, S. N. Pandis, Secondary aerosol formation during the dark oxidation of residential biomass burning emissions. *Environ. Sci. Atmos.* **2**, 1221–1236 (2022).

56. Z. Yang, N. T. Tsona, C. George, L. Du, Nitrogen-containing compounds enhance light absorption of aromatic-derived brown carbon. *Environ. Sci. Technol.* **56**, 4005–4016 (2022).

57. H. K. Lam, R. Xu, J. Choczynski, J. F. Davies, D. Ham, M. Song, A. Zuend, W. Li, Y. L. S. Tse, M. N. Chan, Effects of liquid–liquid phase separation and relative humidity on the heterogeneous OH oxidation of inorganic–organic aerosols: Insights from methylglutaric acid and ammonium sulfate particles. *Atmos. Chem. Phys.* **21**, 2053–2066 (2021).

58. D. J. Losey, R. G. Parker, M. A. Freedman, pH dependence of liquid–liquid phase separation in organic aerosol. *J. Phys. Chem. Lett.* **7**, 3861–3865 (2016).

59. Y. Fan, Q. Li, M. Zhou, J. Sun, S. Ma, T. Xu, Surfactants regulate the mixing state of organic-inorganic mixed aerosols undergoing liquid-liquid phase separation. *Atmos. Chem. Phys.* **25**, 15857–15874 (2025).

60. L. Xu, G. Lin, C. Wu, X. Chen, H. Wang, C. Chen, R. K. Chakrabarty, M. Gao, V. Vakkari, P. G. van Zyl, X. Liu, Strong global radiative effects from wildfire dark brown carbon. *Nat. Geosci.* **19**, 612–619 (2026).

61. X. Wu, Z. Zou, Y. Zhang, Y. Liu, Y. Wang, X. Jiang, Q. Li, X. Wang, K. Huang, G. S. Chelluboyina, R. K. Chakrabarty, A. Laskin, H. Fu, Molecular-specific aromatic compounds absorption drives divergent radiative forcing in high-altitude air and snow over the Tibetan plateau. *Environ. Sci. Technol.* **60**, 5652–5663 (2026).

62. H. Brown, H. Wang, M. Flanner, X. Liu, B. Singh, R. Zhang, Y. Yang, M. Wu, Brown carbon fuel and emission source attributions to global snow darkening effect. *J. Adv. Model. Earth Sys.* **14**, e2021MS002768 (2022).

63. C. L. Friedman, Y. Zhang, N. E. Selin, Climate change and emissions impacts on atmospheric PAH transport to the arctic. *Environ. Sci. Technol.* **48**, 429–437 (2013).

64. D. Calderon-Arrieta, A. C. Morales, A. P. S. Hettiyadura, T. M. Estock, C. Li, Y. Rudich, A. Laskin, Enhanced light absorption and elevated viscosity of atmospheric brown carbon through evaporation of volatile components. *Environ. Sci. Technol.* **58**, 7493–7504 (2024).

65. Z. Liang, L. Zhou, Y. Chang, Y. Qin, C. K. Chan, Biomass-burning organic aerosols as a pool of atmospheric reactive triplets to drive multiphase sulfate formation. *Proc. Natl. Acad. Sci. U.S.A.* **121**, e2416803121 (2024).

66. S. H. Budisulistiorini, M. Riva, M. Williams, J. Chen, M. Itoh, J. D. Surratt, M. Kuwata, Light-absorbing brown carbon aerosol constituents from combustion of indonesian peat and biomass. *Environ. Sci. Technol.* **51**, 4415–4423 (2017).

67. Y. Lin, Y. Han, G. Li, L. Li, X. Zhang, J. Cao, Variability in molecular composition and optical absorption of atmospheric brown carbon aerosols in two contrasting urban areas of China. *Sci. Total Environ.* **926**, 171820 (2024).

68. M. Cheng, M. Kuwata, Development of the low-temperature hygroscopicity tandem differential mobility analyzer (low-T HTDMA) and its application to $(NH_4)_2SO_4$ and NaCl particles. *J. Aerosol Sci.* **168**, 106111 (2023).

69. T. C. Bond, E. Bhardwaj, R. Dong, R. Jogani, S. Jung, C. Roden, D. G. Streets, N. M. Trautmann, Historical emissions of black and organic carbon aerosol from energy-related combustion, 1850–2000. *Global Biogeochem. Cycles* **21**, 840 (2007).

70. L. Giglio, J. T. Randerson, G. R. van der Werf, Analysis of daily, monthly, and annual burned area using the fourth-generation global fire emissions database (GFED4). *J. Geophys. Res. Biogeo*. **118**, 317–328 (2013).

71. L. S. Garca-Coln, L. F. del Castillo, P. Goldstein, Theoretical basis for the Vogel-Fulcher-Tammann equation. *Phys. Rev. B.* **40**, 7040–7044 (1989).

72. R. Zhao, A. K. Y. Lee, L. Huang, X. Li, F. Yang, J. P. D. Abbatt, Photochemical processing of aqueous atmospheric brown carbon. *Atmos. Chem. Phys.* **15**, 6087–6100 (2015).

73. Y. Wang, T. Qiu, C. Zhang, T. Hao, B. R. Go, R. Zhang, M. Gen, M. N. Chan, D. D. Huang, X. Ge, J. Wang, L. Du, R.-J. Huang, Q. Chen, K. I. Hoi, K. M. Mok, C. K. Chan, Y. J. Li, Co-photolysis of mixed chromophores affects atmospheric lifetimes of brown carbon. *Environ. Sci. Atmos.* **3**, 1145–1158 (2023).
